# A PBPK model to evaluate zebrafish eleutheroembryos’ actual exposure: bisphenol A and analogs’ (AF, F, and S) case studies

**DOI:** 10.1007/s11356-022-22741-2

**Published:** 2022-08-31

**Authors:** Pierre-André Billat, Céline Brochot, François Brion, Rémy Beaudouin

**Affiliations:** 1 Experimental Toxicology and Modeling Unit (TEAM), INERIS, Parc ALATA BP2, Verneuil en Halatte, France; 2 Ecotoxicology of Substances and Environments Unit (ESMI), INERIS, Parc ALATA BP2, Verneuil en Halatte, France; 3 UMR-I 02 SEBIO, INERIS, Parc ALATA BP2, Verneuil en Halatte, France

**Keywords:** PBPK model, Zebrafish embryos, *Danio rerio*, Bayesian calibration, Bisphenols, Internal exposure

## Abstract

**Abstract:**

The zebrafish eleutheroembryo model is increasingly used to assess the toxicity and developmental adverse effects of xenobiotics. However, the actual exposure is seldom measured (poorly accessible), while a predictive model could estimate these concentrations. The predictions with a new eleutheroembryo physiologically based pharmacokinetic (PBPK) model have been evaluated using datasets obtained from literature data for several bisphenols. The model simulated the toxicokinetics of bisphenols A (BPA), AF, F, and S through the eleutheroembryo tissues while considering the body and organ growth. We further improved the predictions by adding dynamic flows through the embryo and/or its chorion, impact of experimental temperature, metabolic clearance, and saturation of the absorption by Bayesian calibration. The model structure was determined using the BPA dataset and generalized to the other bisphenols. This model revealed the central role of the chorion in the compound uptake in the first 48 h post-fertilization. The predictions for the BPA substitutes estimated by our PBPK model were compared to available toxicokinetics data for zebrafish embryos, and 63% and 88% of them were within a twofold and fivefold error intervals of the corresponding experimental values, respectively. This model provides a tool to design new eleutheroembryo assays and evaluate the actual exposure.

**Graphical abstract:**

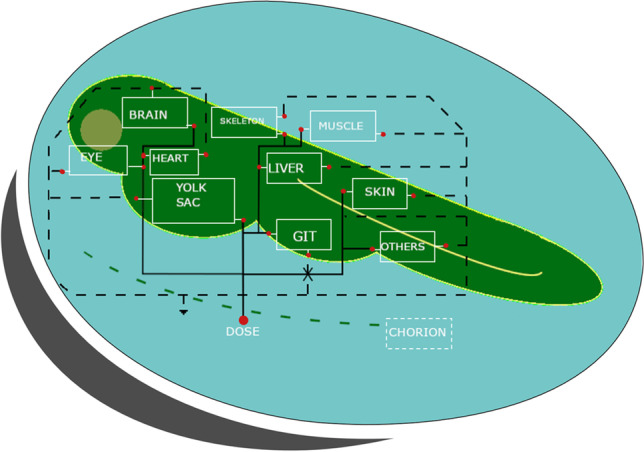

**Supplementary Information:**

The online version contains supplementary material available at 10.1007/s11356-022-22741-2.

## Introduction

The zebrafish eleutheroembryo (zfe) model offers many attractive advantages and has emerged as a relevant alternative vertebrate model in (eco)toxicology. At the regulatory level, specific OECD test guidelines (TG) based on zfe assays have been recently adopted either to determine acute toxicity (fish embryotoxicity assay, TG N°236) (OCDE 2013) or endocrine activity of xenobiotics acting as an agonist of estrogen receptors (EASZY assay, TG 250) (Brion et al. 2019; Pinto et al. 2019). Although these assays provide relevant information on the hazard of xenobiotics, a better characterization of their toxicity or endocrine effects requires accounting for the toxicokinetic processes of the active test substance in the whole organism but also in target tissues (Péry et al. 2013) and even in cells (Billat and Saint-Marcoux 2017). In this regard, it is important to consider the spatio-temporal estimation of internal concentrations in a biological model that is undergoing rapid morphological and physiological changes during the first 120 h post-fertilization (hpf).

New approach methodologies could be complemented to the traditional toxicology to refine the risk-based decision-making approaches and are receiving increased regulatory acceptance (Barton-Maclaren et al. 2022; Punt et al. 2020). In particular, the combination of zfe assays together with a physiologically based pharmacokinetic model (PBPK) allows a better characterization of the actual internal concentrations in an organism following exposure to a chemical (Grech et al. 2017; Nagel 2002; Simeon et al. 2020). PBPK models bridge anatomy, physiology, and biochemical processes to understand and compute xenobiotics’ fate in biological organisms. They estimate xenobiotics’ concentration-time profiles in experimentally inaccessible organs from minimal data, thereby, reducing time, cost, and need for animal experiments (Grech et al. 2017). PBPK models have been developed for the adult zebrafish (Grech et al. 2017; Pery et al. 2014a) and the eleutheroembryo (Simeon et al. 2020). This latter model consists of a series of ordinary differential equations, based on physiological processes (biometrics, ontogeny) and physicochemical processes at steady state, thus assuming instantaneous chemical equilibrium between the exposure medium and the internal tissues. Although relevant, some important morphological/physiological changes that may interfere with the substance uptake, e.g., resorption of the yolk sac and the presence/absence of the chorion (Hagedorn et al. 1997b; Halbach et al. 2020; Schwartz et al. 2021) are not taken into account while they can greatly influence the toxicokinetics (TK) of xenobiotics (Halbach et al. 2020). Therefore, we aimed to refine the existing PBPK model for the zebrafish eleutheroembryo and to assess its relevance, modeling BPA (a widely used environmental pollutant acting as an endocrine disruptor (Beausoleil et al. 2018)), and some of its substitutes, bisphenols AF, AP, B, F, and S (Rosenmai et al. 2014), which have been shown to elicit similar mode of action as BPA notably on the ER-signaling in zebrafish eleutheroembryo (Le Fol et al. 2017a; Pinto et al. 2019).

To do so, an extensive literature search was performed to identify the relevant experimental studies. BPA presents the largest available dataset and, thus, was used to calibrate and test different model structures and to perform a sensitivity analysis of the model. The selected model structure performance was evaluated thereafter on datasets from bisphenol substitutes. Overall, our study successfully improves the existing PBPK model to predict internal concentrations of bisphenols which represents an important step forward for understanding the fate of xenobiotics in the zfe model.

## Methods

### Literature review and data handling

The first step was to perform an extensive literature search to identify experimental studies in which zfe were exposed to bisphenols. Both Pubmed^®^ and ISI Web of knowledge^TM^ databases were used on November the 25th, 2020, using the following request: “abstract =(((zebrafish*) OR (danio*)) AND (bisphenol*) AND ((Juvenile) OR (larva*) OR (embryo*) OR (eleuthero*))).” A total of 209 articles were identified. The papers were selected when (i) at least one concentration was measured both in the embryos, and (ii) the embryo age was no older than 120 hpf at the time of first dosing. Thirteen papers were selected for our study: 10 for BPA (Brown et al. 2019; Fu et al. 2020a; Gibert et al. 2011; Kim et al. 2020; Moreman et al. 2017; Moreman et al. 2018; Saili et al. 2012; Souder and Gorelick 2018; Wu et al. 2017; Yang et al. 2019), 1 for BPAF (Moreman et al. 2017), 2 for BPF (Gibert et al. 2011; Moreman et al. 2017), 3 for BPS (Le Fol et al. 2017b; Moreman et al. 2017; Zhang et al. 2017) (Table S1). One study (Saili et al. 2012) was not included because of uncertainties in the bioanalysis methodology as underlined elsewhere (Souder and Gorelick 2018). In addition, for the BPF, adverse effects seem to occur when zfe are exposed to 25μM BPF (spine deformation), i.e., 2-fold lower than the exposure in the experiment made by Gibert et al. (2011); therefore, a toxicodynamic effect on the TK could not be excluded (Mu et al. 2018). As a result, these data were not used to test the model and were removed from the BPF dataset. Secondly, model predictions were confronted with these data.

To harmonize the datasets, concentrations were expressed in quantity of chemical per body mass. A very low change of zfe total dry mass was observed between days 4 and 6 post-fertilization (lower than 3%) (Bagatto et al. 2001). Therefore, when the information was missing, an average mass of 0.33 mg per eleutheroembryo was used for the conversion (Bagatto et al. 2001; Fu et al. 2020b; Jeffries et al. 2015; Menger et al. 2020).

### PBPK model structure

**Fig. 1:**
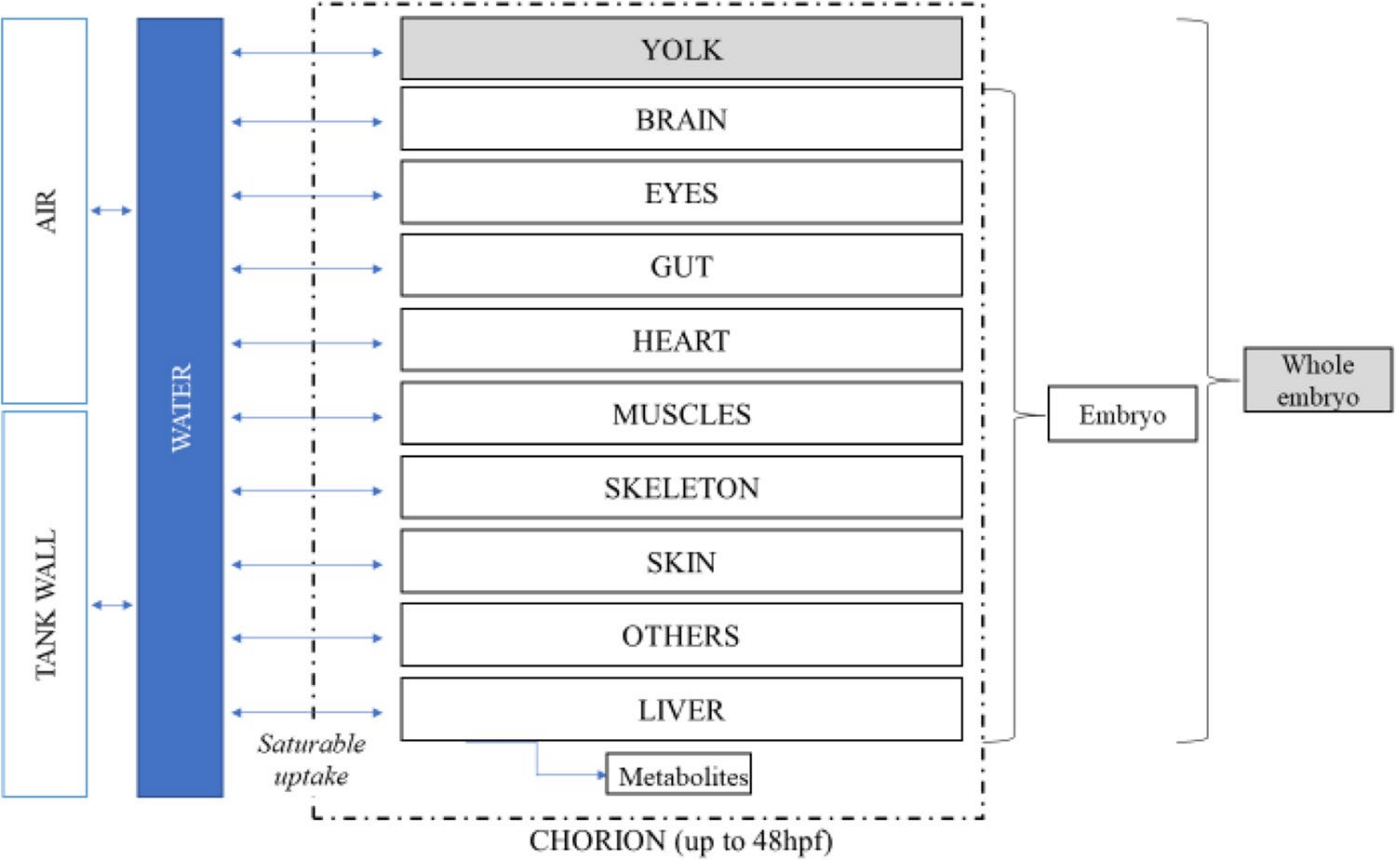
General schematic diagram of the zebrafish eleutheroembryo PBPK including partition to the air and binding to the experimental device walls

Several additions were made to the previously developed PBPK model (Simeon et al. 2020): (i) dynamic efflux from medium to experiment device walls or air, (ii) experimental temperature effect on ontogenesis, (iii) dynamic saturable influx from water to tissues, and (iv) chorion effect on the chemical absorption in the first 48 hpf. Figure 1 presents the schematic structure of the PBPK model that includes ten compartments (yolk, liver, skeleton, gut, eye, brain, heart, skin, muscles, and one lumping the other organs and tissues). Intracellular lysosomes and mitochondria compartments were removed from the initial model to reduce the total number of compartments. Values of the parameters already existing in Simeon et al. (2020) are in Table S2, and the values of the new parameters are presented in Table S3.

The binding of the chemical to the polymer walls (*C*_polymer_, ng/mm^2^) of the experimental device and the evaporation of the compound in the air (*C*_air_, ng/mm^3^) were modeled with dynamic flows:

1 $$\frac{Q_{\mathrm{polymer}}}{dt}={F}_{\mathrm{polymer}}\kern0.5em \times \left({S}_{\mathrm{pw}}\times {C}_{\mathrm{water}}-\kern0.5em \frac{\ {Q}_{\mathrm{polymer}}}{P_{\mathrm{pw}}}\right)$$

2 $${\displaystyle \begin{array}{c}\frac{Q_{\mathrm{air}}}{dt}={F}_{\mathrm{air}}\times {S}_{\mathrm{aw}}\kern0.5em \times \left(\ {C}_{\mathrm{water}}-\kern0.5em \frac{\ {C}_{\mathrm{air}}}{P_{\mathrm{aw}}}\right)\end{array}}$$

where *Q* is the quantity (ng) fixed to polymer or in air, *F*_polymer_ is the exchange rate between water and the polymer (μL/h/mm^2^), *S*_pw_ is the surface of exchanges between polymer and water (mm^2^), *C*_water_ is the concentration in water (mM), *P*_pw_ is the polymer:water partition coefficient (mm^−1^), *F*_air_ is the exchange rate between water and the air (μL/h/mm^2^), *S*_aw_ is the surface of exchanges (mm^2^), and *P*_aw_ is the air:water partition coefficient (no dimension).

Regarding the biometrics, the equations describing the organs’ growth were modified to consider the impact of the experimental temperature using Arrhenius’ formula (detailed in supplementary data, §1. Determination of the Arrhenius temperature). The volumes of the liver, gut, muscle, skeleton, eye, brain, heart, skin, and other tissues were computed as follows:

3 $${\displaystyle \begin{array}{c}{V}_k(t)={V}_{\mathrm{embryo}}(120)\times \left({\exp}^{K_{gk,T}\kern0.5em \times \left(t-{\uptau}_{k,T}\kern0.5em -\kern0.5em {t}_{\mathrm{fec}}\right)}-1\right)\end{array}}$$

4 $${K_{gk}}_T={K_{gk}}_{TR}\times {\exp}^{\left(\frac{T_A}{T_R}-\frac{T_A}{T}\right)}$$

Water temperature also impacts the timing of the organogenesis in addition to the growth rate; hence, the time of growth initiation of the organs was also corrected to integrate the effect of the temperature:

5 $$\frac{1}{\ {\uptau_k}_T}=\frac{1}{\kern0.50em {\uptau_k}_{TR}}\times {\exp}^{\left(\frac{T_A}{T_R}-\frac{T_A}{T}\right)}$$

where *V*_*k*_*(t)* is the volume of the organ *k* at time *t*, *V*_embryo_*(120)* is the volume of the embryo at 120hpf, *K*_*gk*_ is the organ growth rate at the reference temperature, *τ*_*k*_ is the time of growth initiation of the organ *k* (when *t*<*τ*_*k*_, *V*_*k*_*(t)* is null ), and *t*_*fec*_ is the time of fecundation. *TA*, *TR*, and *T* are, respectively, the Arrhenius temperature, the reference temperature of the organ growth (here 298.15 K, corresponding to 25°C), and the temperature of the assay, expressed in Kelvin. The yolk consumption rate constant was also multiplied by Arrhenius’ formula. The total volume of the zfe at time *t* was computed as the sum of the volumes of all the organs and the yolk.

In the initial model (Simeon et al. 2020), the tissue concentrations (*C*_*i*_ concentration in the organ or the yolk *i*, nmol/μL or mm^3^) were assumed to be, at any time, proportional to the concentration in the water (*C*_water_) suggesting an instantaneous equilibrium between the tissues and the medium. However, the actual diffusion is a slower process. To better describe the influx of the chemical in the zfe, a flow was added to mimic the diffusion of the substance. The uptake was assumed to increase with the body surface area from fecundation to the end of the experiment as follows:

6 $${F}_{\mathrm{embryo}:\mathrm{water}}=\kern0.5em {\varphi}_{\mathrm{embryo}:\mathrm{water}}\kern0.75em \times {V_{\mathrm{embryo}}}^{2/3}$$

7 $$\frac{d{Q}_i}{dt}={F}_{\mathrm{embryo}:\mathrm{water}}\times \frac{V_i}{V_{\mathrm{embryo}}}\times \left({C}_{\mathrm{water}}-\frac{{\mathrm{C}}_i}{P_{i:\mathrm{water}}}\right)$$

where *φ*_embryo : water_ (μL/h/mm^2^) is a constant: the exchange rate between the water and the embryo per zfe body surface area. *V*_*i*_ and *V*_embryo_ are the volumes of the organs (μL or mm^3^) and the zfe (including the yolk sac) respectively, *Q*_*i*_ is the quantity in the organ or the yolk (nmol) and *P*_*i* : water_ is the tissue (or yolk):water partition coefficient.

The chorion acts as an additional barrier (Braunbeck et al. 2005; Henn and Braunbeck 2011; Yang et al. 2020); therefore, a change in the flow rates before and after hatching could be expected. To consider this possible change in the exchange rate, a model assuming a discontinuity of the flow rate was compared to the model with a constant exchange rate. The exchange rate was assumed to be linear before hatching (i.e., through the chorion) and then to increase with the body surface area of the zfe after the hatching to the end of the experiment. Hatching was fixed to 48 hpf (Kimmel et al. 1995):

8 $${F}_{\mathrm{embryo}:\mathrm{water}}={\varphi}_{\mathrm{embryo}:\mathrm{water}}^{\mathrm{chorion}}\kern3.5em t<48\ \mathrm{hpf}$$

9 $${F}_{\mathrm{embryo}:\mathrm{water}}=\kern0.5em {\varphi}_{\mathrm{embryo}:\mathrm{water}}\kern0.75em \times {V_{\mathrm{embryo}}}^{2/3}\kern0.75em t\ge 48\ \mathrm{hpf}$$

where ${\varphi}_{\mathrm{embryo}:\mathrm{water}}^{\mathrm{chorion}}$ is the constant of the exchange rates between the water and the embryo through the chorion.

The impact of the chorion on model predictions was then evaluated during the stepwise selection of the model structure (see below).

### Selection of the model structure

To better take into account the animal-physiology and molecule-specific kinetic, three modifications were tested by comparing the model predictions and the observations, namely, the chorion effect on the exchange rate, absence or saturation of the metabolic clearance, and influx saturation with the dose level.

Uptake, metabolism, and disposition of organic xenobiotics have been demonstrated in zfe, highlighting it is metabolically competent, with most of the oxidative processes, esterase, and ADH/ALDH activities starting to occur around 96 hpf (Otte et al. 2017) and expressing fully functional phase I and phase II enzymes at 120 hpf (Jones et al. 2010; Le Fol et al. 2017b; Saad et al. 2017; Verbueken et al. 2017). Furthermore, the metabolic profiles in zfe are qualitatively comparable to that of adults although the metabolic rate was slightly higher in adults as shown for BPS (Le Fol et al. 2017b). As a result, linear or saturable metabolic clearance (i.e., following Michaelis-Menten kinetic) occurring in the liver were compared for each substance using model prediction residues. The parameterization of the two metabolism submodels offers the possibility to detect a marginal impact of the metabolism on the kinetics of the substance (i.e., clearance rates adjusted to a very low value).

10 $$\frac{d{Q}_{\mathrm{liver}}}{dt}={F}_{\mathrm{embryo}:\mathrm{water}}\times \frac{V_{\mathrm{liver}}}{V_{\mathrm{embryo}}}\times \left({C}_{\mathrm{water}}-\frac{C_{\mathrm{liver}}}{P_{\mathrm{liver}:\mathrm{water}}}\right)-\kern0.5em \frac{d{Q}_{\mathrm{met}}}{dt}$$

where *P*_liver : water_ is the liver:water partition coefficient, and the quantity of metabolite *Q*_met_ formed per unit time proportional to the number of liver cells and the liver concentration *C*_liver_ of the parent compound.

When saturable metabolic clearance is assumed:

11 $$\frac{d{Q}_{\mathrm{met}}}{dt}=\frac{V_{\mathrm{liver}}}{V_{\mathrm{liver}\ \mathrm{cell}}}\times {C}_{\mathrm{liver}}\times \frac{V_{\mathrm{max}}\ }{K_M+{C}_{\mathrm{liver}}}$$

where *V*_max_ is the maximum rate of metabolism and *K*_*M*_ is the Michaelis-Menten constant.

Otherwise (linear metabolism):

12 $$\frac{d{Q}_{\mathrm{met}}}{dt}=\frac{V_{\mathrm{liver}}}{V_{\mathrm{liver}\ \mathrm{cell}}}\times {C}_{\mathrm{liver}}\times {K}_{\mathrm{met}}$$

where *K*_met_ is the metabolic clearance.

Finally, to face the wide range of tested dose levels, a saturation process for the tissue uptake of bisphenols was added to the model with:

13 $${\displaystyle \begin{array}{c}\mathrm{Saturation}=\frac{1}{1+\frac{C_{\mathrm{water}}}{{\mathrm{Sat}}_{50}}}\end{array}}$$

14 $${\displaystyle \begin{array}{c}\frac{d{Q}_i}{dt}={F}_{\mathrm{embryo}:\mathrm{water}}\times \frac{V_i}{V_{\mathrm{embryo}}}\times \left(\mathrm{Saturation}\times {C}_{\mathrm{water}}-\frac{C_i}{P_{i:\mathrm{water}}}\right)\end{array}}$$

where Sat_50_ is the half-saturation concentration as described in Bertin et al. (Bertin et al. 2018). This saturation term would provide information on a reduced uptake of bisphenols from water to the internal tissues, depending on the water concentration but independent from the partition coefficient.

To compare the different structures, following the parsimony principle, the model with the lowest Akaike and Bayesian information criteria was retained.

### Parameterization of the model

BPA and its analogs are weak acids, with similar molecular weight ranging from 200.23 to 336.23 g mol^−1^, and very low Henry’s constants suggesting that volatilization from water is not expected; therefore, their concentrations in air were considered null (Table S4).

The parameter values related to the biometrics (size, volume, and mass) and the ontogeny (growth rate, decrease rate, time of onset) were set to the ones previously published (Simeon et al. 2020) (Table S2).

The partition coefficients (*P*_*i*:water_ — i.e., tissue:water — and *P*_pw_) were estimated using the QSAR approach embedded in the VIVD model (Fisher et al. 2019). Because the QSAR models were calibrated using a limited number of compounds in 2D cellular models, we defined a scaling factor (*f*_pc_) to refine the partition coefficient values using information given by the TK observations as previously performed for the zfe (Simeon et al. 2020). Input parameters of the VIVD model were originally informed using zfe organ properties or adult values when zfe data were missing from the literature (Hachicho et al. 2015; Hagedorn et al. 1997a; Pery et al. 2014b).

Before calibration, the set of chemical-specific parameters to be calibrated was identified with a sensitivity analysis (SA) using the variance-based Sobol method (Saltelli et al. 2010; Sobol’ et al. 2007). The sensitivity of the chemical-specific parameters was estimated with uniform distributions ± 10%. In this SA analysis, partition coefficients were set to the values calculated using the VIVD model corrected by the scaling factor (simulation design detailed in Table S5). The influence of the parameters was investigated on several outputs, including total embryo, liver, muscle, and yolk concentration at 24, 48, 72, 96, and 120 hpf. The SA was carried out using the experimental design proposed by the OECD test guidelines of fish embryo toxicity (FET). Details are available in the supporting information document (§4. Supplementary information regarding the parameterization of the model and §5. Simulation details in SI).

A Bayesian approach using MCMC simulations was applied to estimate the other compound-specific parameters from the literature data, i.e., the bisphenol concentrations in the zfe and water: the pre-and post-hatch flow rate constants (${\varphi}_{\mathrm{embryo}:\mathrm{water}}^{\mathrm{chorion}}$ and *φ*_embryo : water_), the absorption saturation constant (Sat_50_), the metabolic clearance (*K*_met_), and the scaling factor (*f*_pc_). The parameters (Sat_50_ and/or *K*_met_) not influencing the compound kinetics were fixed to null or high values. The prior distributions of the estimated parameters were assumed to be uniform (i.e., uninformative, see Table S3). More information regarding the estimation of the model parameters is provided in the supporting information document (§3. Estimation of the model parameters: Bayesian approach and §4. Supplementary information regarding the parameterization of the model in SI).

### Exploring covariate effects on BPA internal concentrations

The maximum posterior values of the final model parameters were used to evaluate the effects of the time course, dose level, and dechorionation on BPA exposure in the zfe. The simulation design was aligned with the OECD test guidelines for the fish embryo toxicity (FET) test N° 236. Simulation details are provided in the supporting information document (§5. Simulation details in SI).

### Evaluation of the model generalizability: BPA substitutes

The BPA model structure and flow rates (pre-and post-hatch) were used to evaluate the model generalizability to other BPA substitutes. The compound-specific parameter, scaling factor of the partition coefficients, was calibrated for each molecule based on chemical concentration data in the zfe and water as their values could not be retrieved from literature data (Table S6).

### Software

All the dynamic model simulations and MCMC calibrations were performed with GNU MCSIM version 6.1.0 (https://www.gnu.org/software/mcsim/) (Bois 2009) and R version 4.0.2. The model code is given as supplemental material (§ Model Code).

## Results and discussion

### PBPK model parameter estimation

**Fig. 2:**
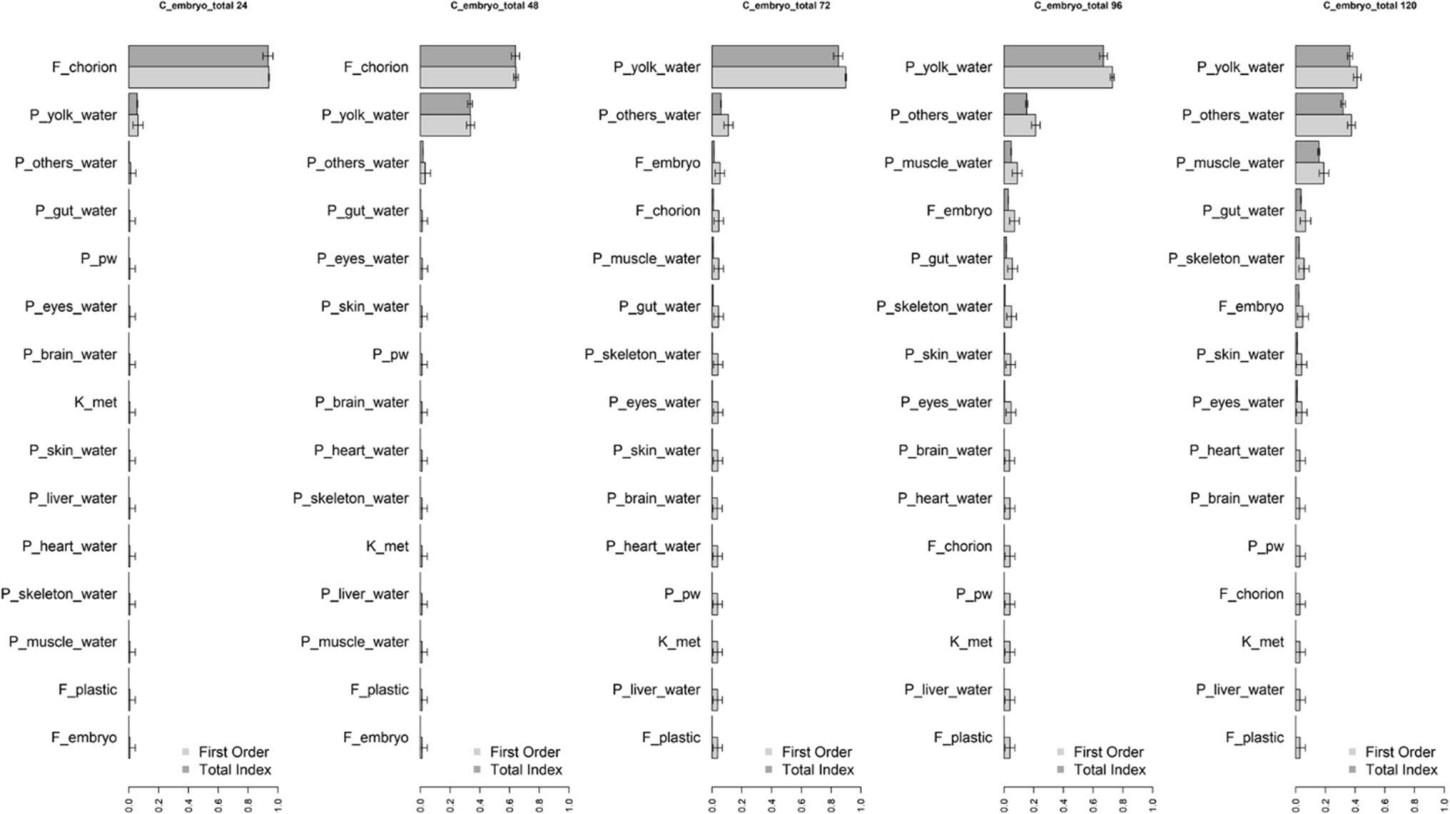
Sensitivity analysis of the model including metabolism (*K*_met_) on total BPA concentrations in zfe (embryo and yolk), at 24, 48, 72, 96, and 120 hpf (from left to right, respectively). The 15 most influential parameters are represented

The PBPK model was calibrated with the BPA dataset to select the most appropriate structure. Before and after the calibration phase on the BPA TK data, a SA of the model was performed (Figs 2, S1A, and S1B).

No data could be found in literature data regarding the concentration of bisphenol in the device walls or the air of capped experiments, while an experiment comparing the bisphenol concentration in a capped on non-capped solution could answer this point. Nevertheless, the objective of this additional step is to determine, as accurately as possible, the water quantity of xenobiotics available for the zfe. In the present case study, the loss of bisphenol is assumed to be mainly due to nonspecific binding to experiment devices (i.e., very low evaporation), depending on the plastic:water exchange surface and, consequently, on the size of the volume of the wells. Therefore, for poorly volatile molecules as the bisphenols of interest, the model could be simplified by removing the equation for volatilization (Eq. 2) or by setting the *P*_aw_ to a very low value. Using this assumption, the simulation of BPA water concentrations was run under every experimental condition of literature (using actual or nominal dose level, renewal of the medium) and the obtained predictions of BPA water concentrations were compared to observed concentrations (Fig. S2).

Table S7 presents the different model structures tested and their AIC and BIC. The addition of the dynamic flows, and thus, no more assuming the instantaneous diffusion, significantly improved the predictions of the model (Table S7). Moreover, the addition of a chorion effect on toxicokinetics (addition of the pre-hatch and post-hatch flows rather than a single flow) improved the predicted curve fit to the observed concentrations. Sensitivity analysis supports this modeling assumption, because it has a strong impact on the zfe total concentration before the hatching (Fig. 2). Therefore, the influence of the chorion could be clearly identified from the TK data available.

The metabolic clearance (*K*_met_) and the absorption saturation constant (Sat_50_), when included in the model, were estimated to values that suggest that these processes do not influence the total BPA concentrations over the range of the tested dose levels (0.438–50,000nM). The current TK data report the total concentration in the zfe only; therefore, metabolism estimation might be uncertain. Indeed, from the SA results, *K*_met_ seems to have a significant impact on the liver concentration only (Fig. 2 and Fig. S1B). In addition, even if the metabolism activity increases with the liver volume (Eq. 12), the impact on the total concentration of the clearance parameter does not increase throughout the time (Fig. 2). Accordingly, exploration of the liver parent or metabolite content would be worthful to assess the metabolic clearance.

The low weight of the metabolism is in line with Gibert et al. (2011) who had not detected metabolites when exposing zfe to 70μM for 72 h (up to 79hpf). Then, according to the principle of parsimony and the sensitivity results, neglecting the metabolism seems reasonable to accurately predict BPA total concentration in the zfe.

Despite the absence of chemical elimination in the zfe, a decrease in BPA concentrations can be observed while the water concentration remains constant and the total amount of BPA in the eleutheroembryo is increasing. Bisphenol apparent clearance could be reasonably explained by tissue volume changes (i.e., dilution by growth: the volume of organs increasing faster (denominator) than the quantity in the tissues (numerator)) and a change in the proportion between organs with a low partition coefficient *P*_*i:*water_, along with the progressive disappearance of the yolk that had a high partition coefficient (simulated organ volume changing over time is presented in Fig. S1 of Simeon et al. (2020)). These two mechanisms are more likely responsible for the concentration decrease rather than metabolism or a “depuration” phase as previously suggested (Sanz-Landaluze et al. 2015; Simeon et al. 2020) even though these mechanisms could not be excluded. Intratissue measurement for BPA would help to evaluate this hypothesis; unfortunately, no concentration data are available at this scale, likely due to the low volume of organs (hard to identify/sample) and the insufficient sensitivity of analytical material.

The most appropriate model for BPA includes the *f*_pc_, and both the pre- and post-hatch flows (Table S7). Table 1 presents the estimated parameters’ values obtained by maximizing the predictive likelihood function (MPV) for this model structure. According to these points, the dose level on BPA kinetic has, as expected, no effect on BPA dose-normalized exposure, and the kinetics is linear with the dose (Fig. S5), which raises the question of an even simpler model. Based on SA results, a simple one-compartment model, considering the zfe as a uniform whole, could not be able to simulate the TK in zfe. Indeed, the key compartments (i.e., those that strongly influence the total concentration) have very different partition coefficients and different time evolutions (e.g., yolk which has a high lipidic content and decreases with time, and liver which is growing and gaining in functionality). Hence, tissue organogenesis strongly influences the total concentration by modifying the ADME process and must be considered in a zfe TK model.

**Table 1:** Summary of posterior distributions for the estimated parameters of the PBPK model for BPA. The distributions were obtained using a Bayesian calibration approach

	Maximum posterior value (MPV)	Median	CI 95%
*f*_pc_	0.365	0.375	[0.276; 0.522]
${\varphi}_{\mathrm{embryo}:\mathrm{water}}$ (μL/h/mm^2^)	8.00	8.40	[5.58; 12.1]
${\varphi}_{\mathrm{embryo}:\mathrm{water}}^{\mathrm{chorion}}$ (μL/h)	0.495	0.499	[0.335; 0.657]
*σ*	2.76	2.84	[2.51; 3.30]

For the BPA, parameter values obtained by calibration show that partition coefficients initially predicted by the VIVD model were corrected using an approximately 1/3 factor (parameter *f*_pc_), indicating an overprediction of the QSAR models (Table S8). The flow parameter distributions seemed to be symmetric, with MPV close to the median values and revealed a narrow credibility interval (CI 95%).

**Fig. 3:**
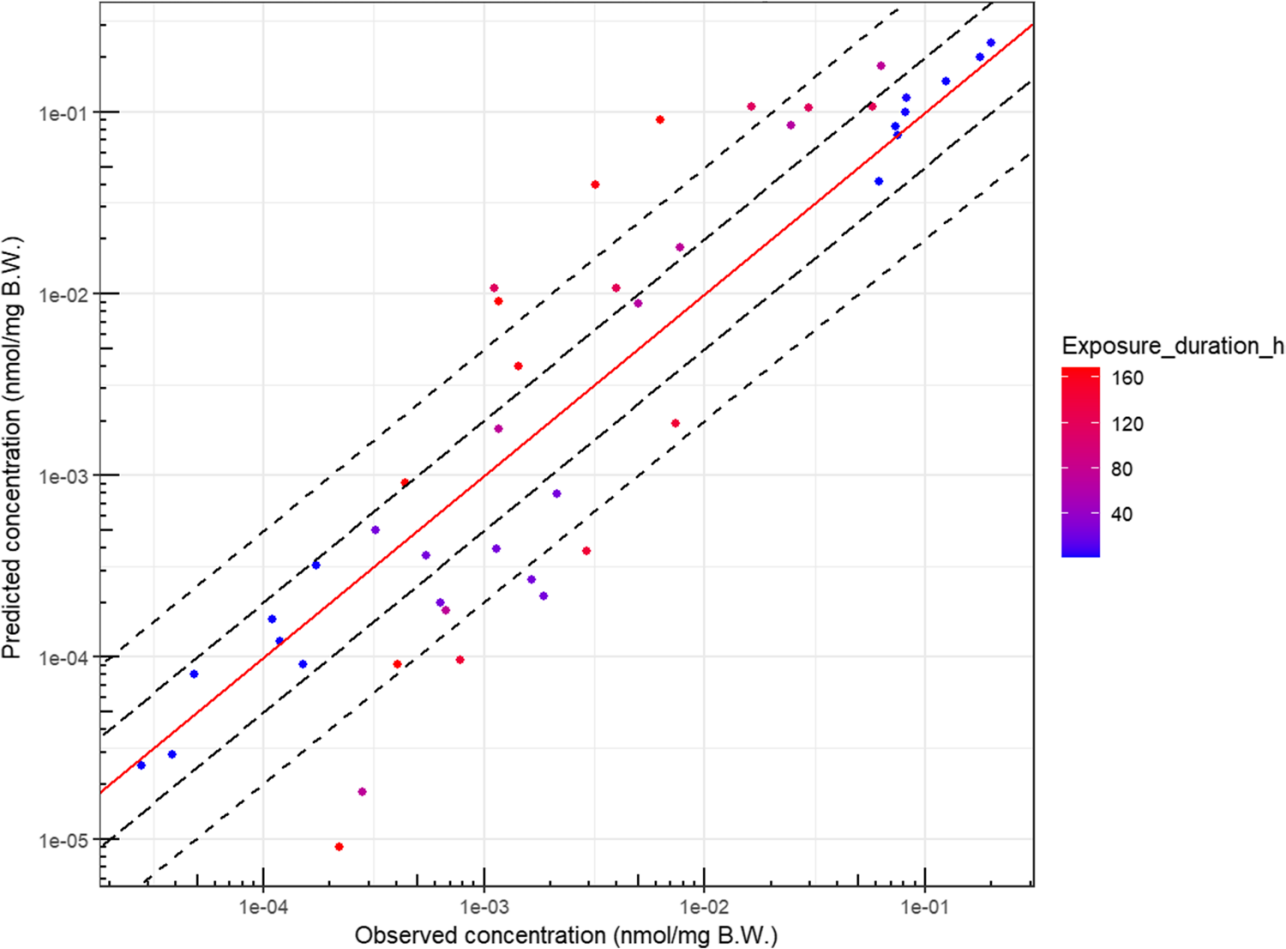
Bisphenol A observed concentrations versus model-predicted concentrations in the zebrafish eleutheroembryo when using the final model. The red line is the line of identity. The long dashed and dashed lines are the 2- and 5-fold errors, respectively

**Fig. 4:**
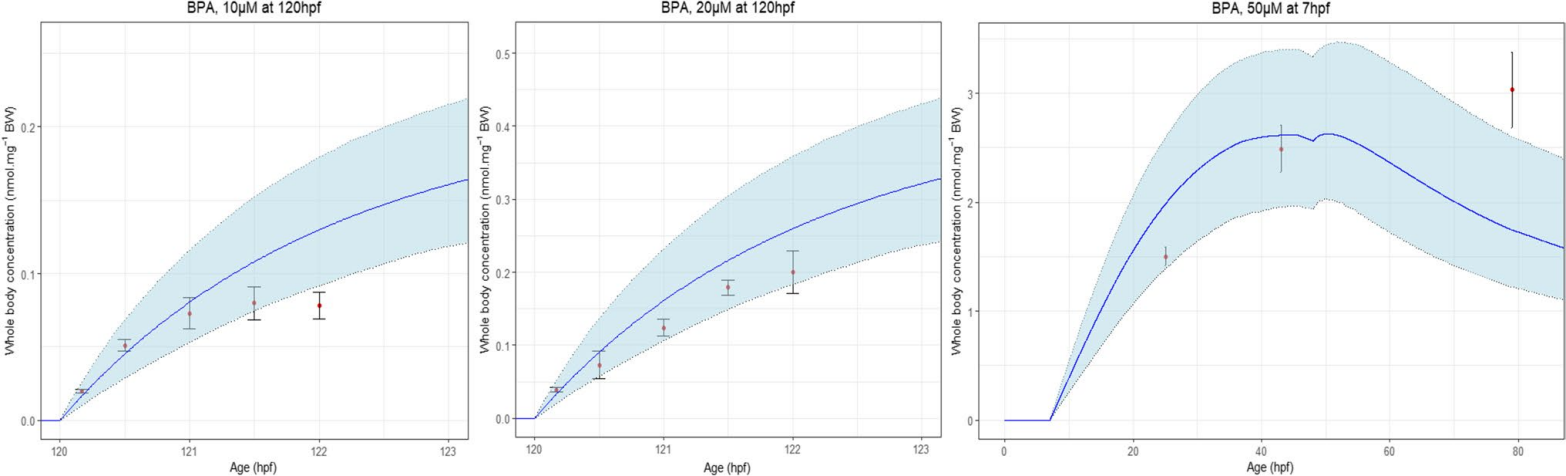
Comparison of the model predicted concentrations (full curve) with the observed concentrations (circles) with standard deviation (error bars) from the BPA data set (the grey area is the 95% credibility interval)

Figures 3 and S2 provide the comparison between the observed and the predicted BPA whole-body concentrations. To compare the simulation results with observed data from a kinetic point of view, experiments including at least 3 timepoints were selected (Fig. 4).

The goodness of fit of the final model showed good model performance with a low bias despite high variability in methodology among literature data: different starting and ending age post-fecundation, different number of zfe, different exposure levels and renewal of the medium, different exposure durations and temperatures, different testing devices, and even different analytical methods. Variability has also been added during the data handling as the data extraction process required the conversion into more exploitable units. With respect to the water concentration predictions (Fig. S2), internal concentrations are less well assessed. This observation suggests that a “simple” steady-state model is not sufficient to explain these discrepancies: in the case of a steady-state model, the internal concentrations would be somewhat proportional to the water concentrations through the whole experiment duration, while here the BPA internal concentrations evolve with time (Gibert et al. 2011; Kim et al. 2020).

The predictions for the BPA TK in zfe show good prediction accuracy however with a narrow credibility interval, even if highest residuals were observed with long-term exposure. Although there is a higher prediction error for these points, no systemic bias could be observed as the internal concentrations are either over- or under-predicted for the last timepoint (−26 and −36 %, A and B respectively, and +30% C). Nevertheless, these long-term exposure residuals suggest also that some underlying mechanisms are not included in the model. This discrepancy could be due to some neglected biological processes, such as but not restricted to, progressive permeabilization and nonspecific binding to the chorion, gills increase in functionality (i.e., gill rudiments appear around 72hpf and 30–50% are vascularized at 168hpf (Shadrin and Ozernyuk 2002)), other sites of metabolism than the liver, or development of the systemic circulation (draft starting at 24hpf).

Regarding absorption, the main route was assumed to be transcutaneous, and the gill absorption was a minor route that could be neglected. This assumption sounds reasonable, because small fish species have a large cutaneous surface-area-to-volume ratio, and the skin is almost as thin as the gill (Lien et al. 1994). Accordingly, in fish larvae as zfe, skin surface area exceeds that of the gills which are fully functional only at 96 hpf (Lien et al. 1994; Wingert et al. 2007). Nevertheless, quantification of the various routes of absorption/excretion in future research would lower uncertainty in zfe PBTK models, in particular the relative contribution of the skin to the overall absorption and excretion of chemicals.

Another source of uncertainty is the possible occurrence of adverse effects on physiology at the highest tested dose levels that may interfere with ADME processes. Indeed, the BPA lowest adverse effect dose levels are 50μM before hatching and 25μM after hatching (at which pericardial and yolk sac oedemas, tail, and spine malformations were observed) (Scopel et al. 2020). These values are similar to or lower than the two highest exposure included in this study (Gibert et al. 2011; Moreman et al. 2017). However, the highest internal concentration predictions were close to the observed internal concentrations suggesting that, at the tested dose levels, the toxicodynamics have no or low effect on the TK (Figs 3 and S3) and no dose effect was observed in diagnostic plots (Fig. S4).

Extrapolation from BPA exposure in zfe to exposure in adult zebrafish seems complex. PBPK models have been also applied to BPA in adult zebrafish (Grech et al. 2017; Pery et al. 2014b) and recently precisely adapted to BPA TK in different adult fish models (Mit et al. 2022). These models highlight the central role of gills absorption, a rapid and extensive metabolism, and the excretion of the metabolites by the bile in the adult zebrafish, while these processes have less impact on the zfe.

### Predicting covariate effects on BPA internal concentration

The calibrated model was used to evaluate the impact of age and chorion on BPA exposure.

**Fig. 5:**
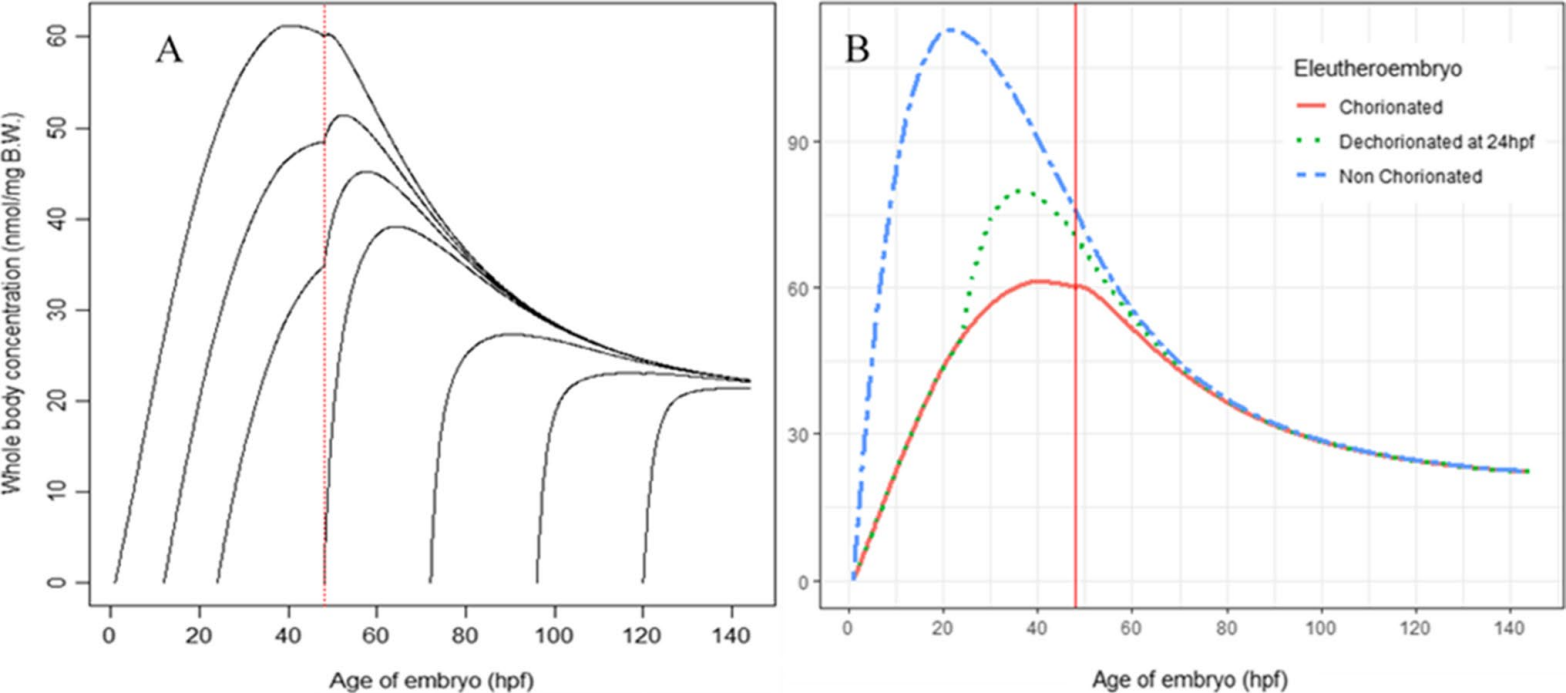
Simulations of the impact of covariates on BPA exposure (**A** impact of the age of treatment, **B** impact of the chorion) in zebrafish eleutheroembryos exposed to 1 μM of BPA for 144 h (vertical red line is the hatching at 48 hpf)

The effect of the initial age on BPA exposure was explored using our calibrated model, by simulating the treatment at different ages post-fecundation (Fig. 5A, Table S9). The predicted maximal concentration (*C*_max_) decreased with age by a 3-factor, from the earliest treatment (1hpf) to the last treatment (120hpf). The time post-dosing to reach *C*_max_ (*t*_max_) slightly decreased during the pre-hatching period (from 39.5 to 38.9 h post-dosing), while it increased after hatching (from 16.3 to 22.0 h post-dosing). This result is in line with the physiological changes occurring during the zfe development: the permeability of the zebrafish slightly changes during the pre-hatching period with a permeability that is increasing from the 6-somite stage (c.a. 12 hpf) (Hagedorn et al. 1997b; Harvey et al. 1983), being higher around hatching (Harvey and Chamberlain 1982) and then the internal drug exposure depends on the growth and maturation of the organs.

In our model, the BPA *C*_max_ decreased significantly with age, suggesting that, the earlier the zfe are exposed, the higher the exposure. Therefore, the present model would help to design further toxicology studies by suggesting the age and duration of exposure and to reach the targeted internal dose.

Our model described that no steady state could be observed for this molecule. Therefore, bioconcentration factors (BCF) could not be accurately estimated from the zfe in vivo kinetics when using a custom experimental design. This finding could, at least partially, explain the wide range of observed BCF found in zebrafish embryo literature, with a BPA BCF ranging from 1.44 to 643, depending on the initial concentration, duration exposure, and age of embryo (Brown et al. 2019; Gibert et al. 2011; Kim et al. 2020; Moreman et al. 2017; Wu et al. 2017).

As some studies dechorionated zfe, the impact of this approach was also explored (Fig. 5B). The dechorionation has a clear effect on BPA internal exposure. Whole-body exposure to BPA was higher in the non-chorionated eleutheroembryo and lower in the chorionated eleutheroembryo. This is in line with literature data as the chorion protects the eleutheroembryo from exposure to xenobiotics during the early development, by decreasing the flow towards the eleutheroembryo and acting as a semipermeable membrane up to 48hpf (Henn and Braunbeck 2011; Yang et al. 2020). BPAF, for instance, is more toxic in dechorionated zfe than in chorionated zfe, suggesting that the chorion plays a protective role with regard to this chemical family (Yang et al. 2020). The reduction in the flow might be not due to the size of the tested compound as a previous study shows reduced uptake starting at 2000 g mol^−1^ and no uptake at 4000 g mol^−1^ for PEG (here the maximal MW is 336.23 g mol^−1^) (Pelka et al. 2017), but maybe rather due to the ionic charge of these compounds (Brox et al. 2016).

The effect of the chorion does not suddenly disappear after hatching but tends to decrease with time as, at around 66 hpf (i.e., 65 h post-dosing), no clear difference in the whole-body concentrations could be evidenced (less than 5% difference within each group).

In the present study, the hatching was assumed to occur at 48 hpf. Considering the variability of the experimental conditions in the included studies, it would be of interest to set the hatching time as a parameter to be estimated by the model and per molecule and experiment in future updates. Indeed, to the biological intragroup variability, intergroup variability in hatching time can be observed as some molecules could directly interfere with the hatching process (Yang et al. 2020). Thus, exploring the concentrations around 48 hpf may be puzzling, giving a bimodal distribution of the whole-body concentrations (i.e., one distribution for the hatched and another one for the non-hatched eleutheroembryos).

### Evaluation of the model generalizability: BPA analogs

**Fig. 6:**
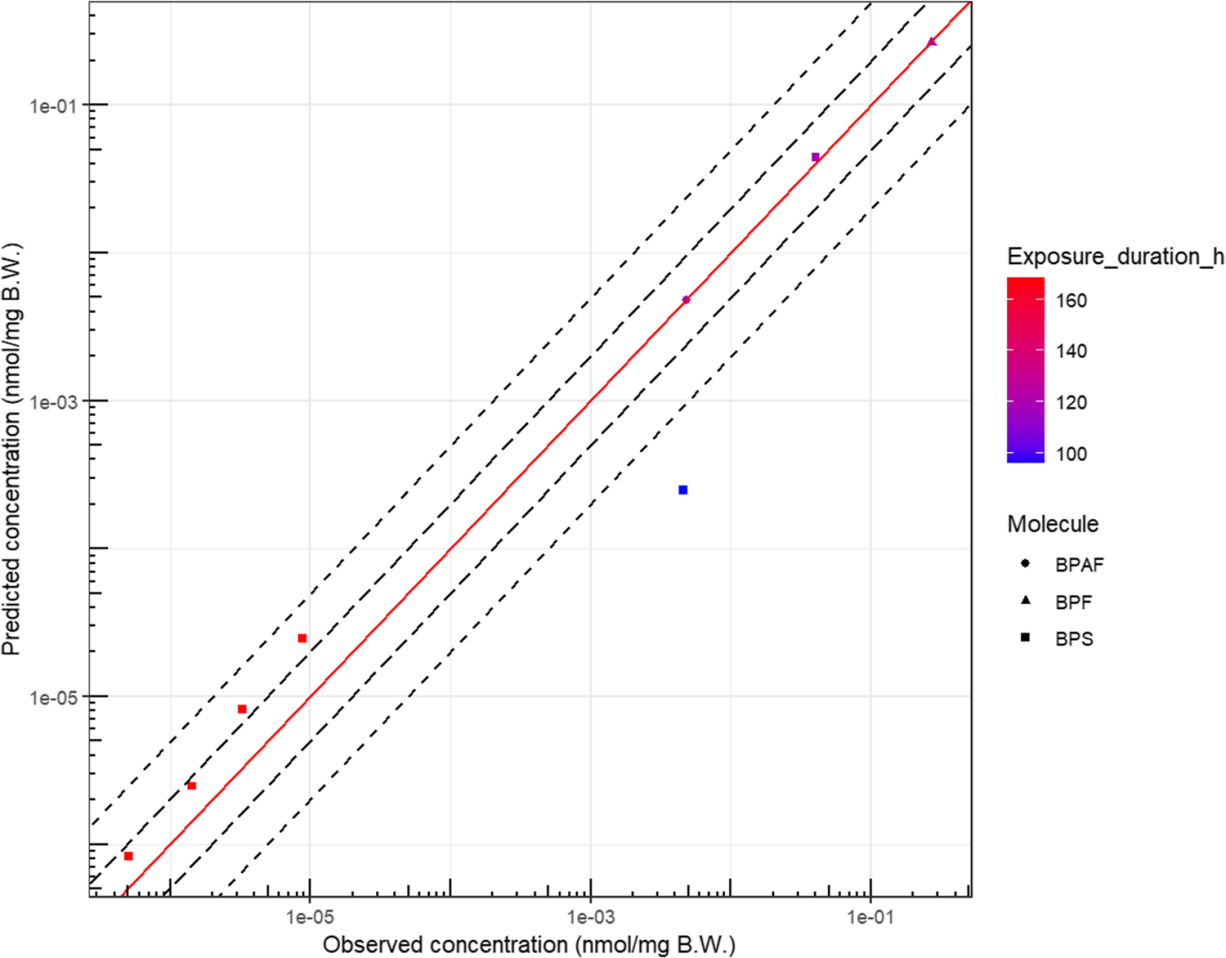
Observed versus model-predicted concentrations of BPAF, BPF, and BPS in the zebrafish eleutheroembryo in water for bisphenol AF, F, and S when using the PBPK model. The red line is the line of identity. The long dashed and dashed lines are the 2- and 5-fold errors, respectively

Since few data are available for the other bisphenols and the chemical structure and properties are close among all the bisphenols, the flow rates water/chorion/embryo (pre-hatch) and water/embryo (post-hatch) were fixed to the values obtained for BPA (i.e., 0.495μL/h and 8.00μL/h/mm^2^, respectively). However, some slight differences in diffusion parameters could exist between each form; then, further in vitro experiments using these analogs would be helpful to determine more accurately these parameters. Here, the chorion crossing was considered to be similar among all of the bisphenols due to their structural similarities. The predictions obtained using the flow between water/chorion/embryo initially calibrated using the BPA data give accurate predictions for the other bisphenols (Figs 6 and S6). Consequently, despite a low number of studies, it seems that the flow rates for molecules of comparable structures and physicochemical properties are similar.

Only the partition coefficient corrector (*f*_pc_) and geometric standard deviation (*σ*) were calibrated for the analogs since they are compound-specific parameters (Table S5, §4. Supplementary information regarding the parameterization of the model). The posterior distributions for the updated parameters are presented in Table 2.

**Table 2:** Summary of posterior distributions for parameters updated from prior estimates using the Bayesian-PBPK models for the substitutes’ dataset

	MPV	Median	CI 95%
BPAF			
*f*_pc_	1.64E^−2^	1.74E^−2^	[1.21E^−2^; 3.51E^−2^]
*σ*	1.07	1.31	[1.09; 1.86]
BPF			
*f*_pc_	1.70	2.24	[1.19; 8.09]
*σ*	1.14	1.64	[1.12; 3.56]
BPS			
*f*_pc_	0.114	0.131	[6.98E^−2^; 0.258]
*σ*	2.36	2.58	[2.06; 3.51]

The tissue:water partition coefficient corrected using the *f*_pc_ are summarized in Table S7. The VIVD model is a semiempirical QSAR approach that requires several physicochemical properties such as logP, Henry’s constant, pKa, and acidic/neutral/basic form but no in vitro data. The scaling factor significantly differs among the various bisphenols and is generally lower than 1, suggesting that the initial values estimated by the QSAR model (prior values) are overpredicted. Therefore, the global scaling factor (*f*_pc_) allows refining the value of the partition coefficients with the TK data obtained in ZFE (posterior values, update of the parameter values with new information).

The corrected partition coefficients (*P*_*i*:water_) coefficients of each bisphenol were in the same order of magnitude except for BPS. Indeed, BPS has a lower log *K*_ow_ and a higher solubility than the other tested bisphenols, suggesting that the observed discrepancy in partition coefficients would be due to different physicochemical behaviors.

The whole-body concentrations of the 3 xenobiotics estimated by our PBPK model were compared to available TK data for zebrafish embryos (Fig. 6).

On a total of 8 data points, 5 and 7 were within a factor of 2 and 5, respectively. Due to the low number of observations, no external validation (i.e., using the model with the calibrated parameters on external data) could be performed.

The data heterogenicity is a known source of bias when modeling, but it could also give precious information about the model’s robustness. In the substitute dataset, the outlier value corresponds to the data obtained with radiolabeled BPS and radio-HPLC measurement (Le Fol et al. 2017b), while other studies were based on mass spectrometry analysis. This difference in analytical methods could, at least partially, explain the discrepancy between predicted and observed concentrations.

**Fig. 7:**
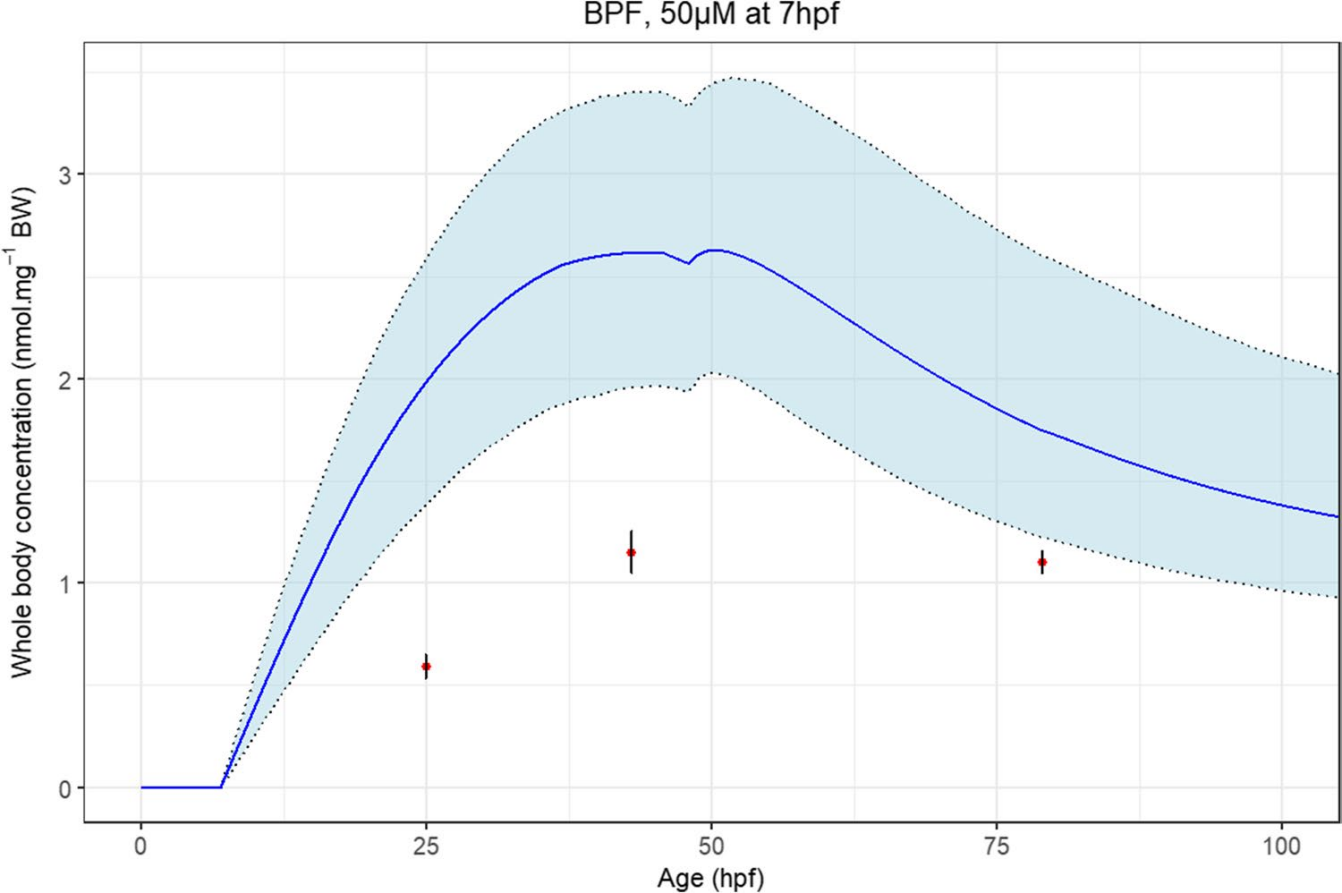
Comparison of the model predicted concentrations (full curve) with the observed concentrations (triangles) with standard deviation (error bars) from the BPF data set (the grey area is the 95% credibility interval)

Figure 7 presents the observed BPF concentrations in the zfe and the simulated kinetics for the study, previously excluded for the parameter calibration step, including at least 3 timepoints post-dose (Gibert et al. 2011).

Saturation processes may occur in BPF, as the fit to the data was significantly improved when adding this parameter (Fig. S7). The saturation constant was then estimated to be 19.0 μM by Bayesian calibration. As no saturation was observed with the other tested compounds, BPF seems to have either lower uptake saturation constant, and/or that BPF toxicodynamics impacted the uptake of this compound. Indeed, when added to the model, calibration results of both saturation and metabolism parameters (BPAF and BPS) or just metabolism (BPF) tend to null values. Thus, under the experimental conditions of the included studies, these parameters do not seem to influence greatly the fate of bisphenols in zfe while it could not be excluded that there is saturation or metabolic clearance of these compounds occurring in zfe (BPF metabolism was observed but not quantified in Gibert et al. (2011) for instance). Dedicated ADME experiments of bisphenol should be performed to settle this finding.

**Fig. 8:**
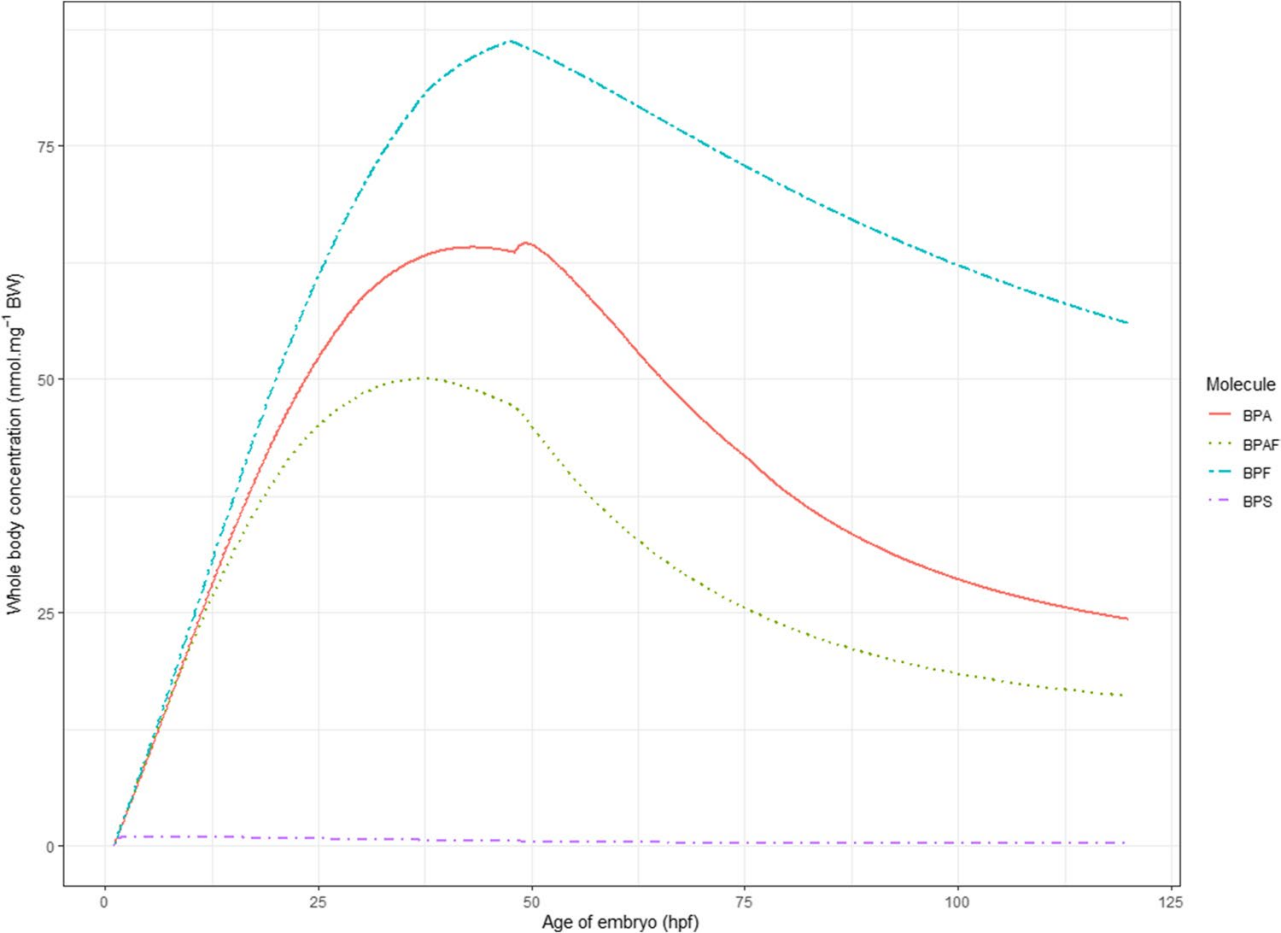
Predicted concentrations versus time profiles of BPA, BPAF, BPF, and BPS in zebrafish eleutheroembryo incubated in 1μM at 1hpf

A comparison of the model predictions for the BPA, BPAF, BPF, and BPS (simulation inputs were parameterized according to the ad hoc OECD test guidelines for zfe toxicity) clearly shows that zfe internal exposure to BPF was higher than exposure to BPA (about + 30–50%) and exposure to BPF (about + 70–100%), while the internal exposure to BPS was lower than 2% of the exposure to other analogs (Table S9, Fig. 8). These different toxicokinetic profiles could, at least partly, explain the differential toxicity and estrogenic responses reported in zfe exposed to the bisphenols (Le Fol et al. 2017a; Moreman et al. 2017; Pinto et al. 2019). As an illustration, Moreman et al. (2017) demonstrated that the highest estrogenic activity was observed with BPAF, then BPA and BPF, and that BPS implies the lowest estrogenic response of the four tested compounds (Moreman et al. 2017). In this regard, the predicted pharmacokinetics and the observed responses reported in zfe were slightly different for the bisphenols investigated in this study. This fact highlights the need to further improve the present model by linking TK with toxicodynamics in zfe. In this perspective, prediction of intratissue concentrations of xenobiotics (for instance, by taking advantage of recent developments in imaging mass spectrometry (Halbach et al. 2019)) would help to better predict the tissue-specific responses (e.g., brain aromatase induction as measured in the EASZY assay).

The present model includes several features enabling the prediction of saturation and metabolic processes, and low permeability of the chorion to heavy molecular mass molecules, but it would be also advisable to investigate to which extent the refined zfe-PBPK model can be generalized to other classes of xenobiotics.

Notwithstanding, this updated PBPK model provides a relevant tool to design eleutheroembryo assays and to better apprehend the fate of xenobiotics in these assays thereby reinforcing the interest of the zfe model and related assays (OCDE 2013, 2021) for the hazard assessment of xenobiotics.

## Data Availability

The datasets of the current research can be available from the corresponding author after a reasonable request.

## Supporting information

Details of parameterization and simulation methods, properties of the test compounds, additional modeling results, and model code. ESM 1 (DOCX 1096 kb)
