# Correction to: A PBPK model to evaluate zebrafish eleutheroembryos’ actual exposure: bisphenol A and analogs’ (AF, F, and S) case studies

**DOI:** 10.1007/s11356-022-22994-x

**Published:** 2022-09-10

**Authors:** Pierre‑André Billat, Céline Brochot, François Brion, Rémy Beaudouin

**Affiliations:** 1 Experimental Toxicology and Modeling Unit (TEAM), INERIS, Parc ALATA BP2, Verneuil en Halatte, France; 2 Ecotoxicology of Substances and Environments Unit (ESMI), INERIS, Parc ALATA BP2, Verneuil en Halatte, France; 3 UMR-I 02 SEBIO, INERIS, Parc ALATA BP2, Verneuil en Halatte, France

**Correction to: Environmental Science and Pollution Research**

**https://doi.org/10.1007/s11356-022-22741-2**

The article A PBPK model to evaluate zebrafish eleutheroembryos’ actual exposure: bisphenol A and analogs’ (AF, F, and S) case studies, written by Pierre‑André Billat, Céline Brochot, François Brion and Rémy Beaudouin, was originally published electronically on the publisher’s internet portal on 31 August 2022 without open access. With the author(s)’ decision to opt for Open Choice the copyright of the article changed on 07 September 2022 to © The Author(s) 2022 and the article is forthwith distributed under a Creative Commons Attribution 4.0 International License, which permits use, sharing, adaptation, distribution and reproduction in any medium or format, as long as you give appropriate credit to the original author(s) and the source, provide a link to the Creative Commons licence, and indicate if changes were made. The images or other third party material in this article are included in the article’s Creative Commons licence, unless indicated otherwise in a credit line to the material. If material is not included in the article’s Creative Commons licence and your intended use is not permitted by statutory regulation or exceeds the permitted use, you will need to obtain permission directly from the copyright holder. To view a copy of this licence, visit http://creativecommons.org/licenses/by/4.0.

The Original article has been corrected.

