# Supplementary material for: A PBPK model to evaluate zebrafish eleutheroembryos’ actual exposure: bisphenol A and analogs’ (AF, F, and S) case studies
Source: Environ Sci Pollut Res Int. 2022 Aug 31;30(3):7640–53. doi: 10.1007/s11356-022-22741-2 (PMC9894996; doi:10.1007/s11356-022-22741-2)
Supplement: ESM 1 — (DOCX 1096 kb) [file 11356_2022_22741_MOESM1_ESM.docx]

SUPPORTING INFORMATION

A PBPK model to evaluate zebrafish eleutheroembryos actual exposure: bisphenol A and analogs (AF, F and S) cases studies

Pierre-André Billat^a^, Céline Brochot^a^, François Brion^b,c^, Rémy Beaudouin^a ,c^ .

^a^ INERIS, Experimental toxicology and modeling unit (TEAM), Parc ALATA BP2, Verneuil en Halatte, France

^b^ INERIS, Ecotoxicology of substances and environments unit (ESMI), Parc ALATA BP2, Verneuil en Halatte, France

^c^ UMR-I 02 SEBIO, Parc ALATA BP2, Verneuil en Halatte, France INERIS

Total page number 37, including ten tables, eight figures and the model code

**Table of contents**

[1. Determination of the Arrhenius temperature 2](#_Toc109997653)

[2. Sensitivity analysis 3](#_Toc109997654)

[3. Estimation of the model parameters: Bayesian approach 3](#_Toc109997655)

[4. Supplementary information regarding the parameterization of the model 3](#_Toc109997656)

[5. Simulation details 4](#_Toc109997657)

[6. Additional tables 5](#_Toc109997658)

[7. Additional figures 14](#_Toc109997659)

[8. Model code 22](#_Toc109997660)

[9. References 36](#_Toc109997661)

Determination of the Arrhenius temperature

Kooijman described how the temperature affects the metabolism rate in most species and proposed the Arrhenius relationship to describe the effect of temperature on biological processes([Kooijman &Kooijman 2010](#_ENREF_10)):

$R_{T}= R_{TR}\times{exp}^{(\frac{T_{A}}{T_{R}}-\frac{T_{A}}{T})}$(1, SI)

R_T_ and R_TR_ are the physiological rates at the water temperature T (in Kelvin) and the reference temperature T_R_ (~~equal to 298.15~~ in Kelvin), T is the water temperature of the experiment (in Kelvin), and T_A_ is the Arrhenius temperature which governs the curve of the temperature-response relationship (in Kelvin).

The equation (1, SI) was calibrated by replacing the rates (1/time) with the inverse of the developmental of the stages of embryonic development reported by [Kimmel et al. (1995)](#_ENREF_9)~~, the~~ under different water temperatures (“developmental time”): ~~equation becomes~~:

$\frac{1}{Developmental time\left( T \right)}= \frac{1}{Developmental time\left( T_{R} \right)}\times{exp}^{(\frac{T_{A}}{T_{R}}-\frac{T_{A}}{T})} ($2, SI)

The reference temperature T_R_ was fixed to 298.15K (organ growth was calibrated to this temperature by Siméon et al. (2020)). Arrhenius temperature (T_A_) was calibrated using the developmental times of the stage “Hatching” described by [Kimmel et al. (1995)](#_ENREF_9) (easily observable, defined in Table 1 in Kimmel et al. 1995) at different temperatures (298.15, 301.65, 306.15K). Using the least-squares regression method of the Arrhenius relationship on the observed data, T_A_ was estimated to be 6930K (Fig S8).

Sensitivity analysis

The sensitivity analysis was carried out on the exposure scenario advised in the OECD FET guideline (see §5. Simulation details, SI) and using the variance-based Sobol method ([Saltelli et al. 2008](#_ENREF_15), [Sobol et al. 2007](#_ENREF_17)). The influence of 19 parameters (presented in Table S1) was investigated on model outputs (e.g., concentration in blood, liver, embryo, and total embryo) at 24, 48, 72, 96, and 120 hpf. Partition coefficients were calculated using the VIVD method described in ([Fisher et al. 2018](#_ENREF_2)), [Grech et al. (2019)](#_ENREF_6).

Two different analyses were performed: (i) the first analysis was performed on the initial model, including correcting factor of the partition coefficients (fpc), metabolism (Kmet), and saturation process (Sat50) (Figure S1A), and (ii) the second analysis was performed on the final model (PC corrected by the fpc and saturation process not included in SA) (Figure 2 and Figure S1B).

Estimation of the model parameters: Bayesian approach

The zfe PBPK model was fitted to the experimental data (*i.e.*, the concentration of bisphenols in zfe) in a Bayesian framework to estimate the main parameters of the PBPK model. This approach reveals numerous advantages such as considering both variability and uncertainty([Gelman &Rubin 1996](#_ENREF_4)). This tool is useful to update the available knowledge about parameters (θ) of previous models by using observed data (y)([van de Schoot et al. 2021](#_ENREF_19)). Briefly, the Bayesian theorem states that the *a posteriori* probability distribution P(θ|y) is proportional to an a priori P(θ), (prior) multiplied by the likelihood, *i.e.* the probability to predict the data at each time point and under each specific experimental conditions given model parameters (and structure) P(y|θ). From the *a priori* distribution and given the model parameters, the posterior distributions were generated using the Markov chain Monte Carlo technique. Finally, the values of estimated priors $\hat{\theta}$ for which the likelihood value is maximized were selected and referred to as maximum posterior values (MPV).

Supplementary information regarding the parameterization of the model

The data on C_water_ and C_embryo_ were assumed to be log-normally distributed around the model predictions (taken as geometric mean) with the geometric standard deviation σ. The σ was also estimated and assumed to be *a priori* distributed normally around 1.5 $\pm$ 1.5 SD with a truncation from 1.1 to 10 (*i.e.* between 10% error and a 10-fold error at most). Three Markov chains of 10,000 iterations were simulated for each chemical. The convergence of the three chains was diagnosed by the estimated scale reduction using Gelman and Rubin’s convergence criterion([Gelman &Rubin 1996](#_ENREF_4)) and recommended being lower than 1.05. The posterior distribution of the selected parameters was assessed in 3,333 last iterations of each chain for each molecule (i.e. about 10,000 iterations in all).

Simulation details

Briefly, one eleutheroembryo per well was exposed to the chemical in 2mL of the exposure solution. The testing device consisted of a 24-well low-binding plate (internal diameter of 20 mm and capacity of 5 mL) kept at 26°C.

To explore the embryo age effect on BPA exposure, the treatment was simulated at different ages post fertilization: 1 hpf (as advised in the OECD FET guideline), 6 hpf, 12 hpf, 24 hpf, 48 hpf, 72 hpf, 96 hpf, and 120 hpf. In all simulations, each zfe was exposed to 1 µM of BPA up to 144 hpf.

To explore the effect of the dose level on BPA kinetic, a 96-hour exposure was simulated in zfe aged 1 hpf at 4 concentrations: 1, 10, 100, and 1,000 µM.

The impact of the chorion on the toxicokinetics was assessed by simulating a 96-hour exposure (1 µM BPA, 1 hpf) under three different conditions: in chorionated zfe, non-chorionated zfe, and zfe dechorionated at the age of 24 hpf, as described in literature([Henn &Braunbeck 2011](#_ENREF_7)).

Additional tables

**Table S1.** Toxicokinetic studies of bisphenols selected for PBPK modeling.

| Molecule | Nominal water concentrations (nM) | Number of timepoints per dose level | Number of eleutheroembryos used per timepoint | Age at the time of dosing (hpf) | Exposure duration  (hours) | Temperature  (K) | Reference |
| --- | --- | --- | --- | --- | --- | --- | --- |
| BPA | 0.438 – 4.38– 43.8 - 438 – 4,380 | 2 | 40 | 6 | 166 | 301.15 | ([Wu et al. 2017](#_ENREF_20)) |
|  | 5 -20 | 2 | 10 | 48 | 24 | 301.65 | ([Souder &Gorelick 2018](#_ENREF_18))* |
|  | 10 | 2 | 10 | 6 – 24 – 48 -72 - 96 | 24 | 301.65 | ([Souder &Gorelick 2018](#_ENREF_18))* |
|  | 17.5 – 87.6 | 1 | 100 | 2 | 142 | 301.15 | ([Fu et al. 2020](#_ENREF_3)) |
|  | 219 – 2,190 | 2 | 2 | 6 | 162 | 301.15 | ([Yang et al. 2019](#_ENREF_21)) |
|  | 438 – 4,380 | 1 | 20 | 3 | 117 | 301.15 | ([Moreman et al. 2017](#_ENREF_12)) |
|  | 438 – 4,380 | 1 | 120 | 6 | 96 | 301.15 | ([Brown et al. 2019](#_ENREF_1)) |
|  | 10,000 – 20,000 | 5 | 20 | 120 | 2 | 300.65 | ([Kim et al. 2020](#_ENREF_8)) |
|  | 43,800 | 1 | 20 | 0 | 120 | 301.15 | ([Moreman et al. 2018](#_ENREF_13)) |
|  | 50,000 | 3 | 60 | 7 | 72 | 301.65 | ([Gibert et al. 2011](#_ENREF_5)) |
| BPAF | 297 | 1 | 20 | 3 | 117 | 301.15 | ([Moreman et al. 2017](#_ENREF_12)) |
| BPF | 4,990 | 1 | 20 | 3 | 117 | 301.15 | ([Moreman et al. 2017](#_ENREF_12)) |
|  | 50,000 | 3 | 60 | 7 | 72 | 301.65 | ([Gibert et al. 2011](#_ENREF_5)) |
| BPS | 4 -12 – 40 – 120 | 1 | 500 | 4 | 140 | 301.15 | ([Zhang et al. 2017](#_ENREF_22)) |
|  | 1,000 | 1 | 60 | 0 | 96 | 301.15 | ([Le Fol et al. 2017](#_ENREF_11)) |
|  | 200,000 | 1 | 20 | 3 | 117 | 301.15 | ([Moreman et al. 2017](#_ENREF_12)) |

* dechorionation was performed before the exposure

**Table S2.** Physiological parameter values of the zebrafish embryo model.

| Parameters | Symbols | Units | Values | References |
| --- | --- | --- | --- | --- |
| Initial organ growth time |  |  |  |  |
| Brain | τ_brain_ | hours | 9 | ([Simeon et al. 2020](#_ENREF_16)) |
| Eyes | τ_eyes_ | hours | 11 | ([Simeon et al. 2020](#_ENREF_16)) |
| Gut | τ_gut_ | hours | 10 | ([Simeon et al. 2020](#_ENREF_16)) |
| Heart | τ_heart_ | hours | 30 | ([Simeon et al. 2020](#_ENREF_16)) |
| Liver | τ_liver_ | hours | 16 | ([Simeon et al. 2020](#_ENREF_16)) |
| Skeleton | τ_skeleton_ | hours | 48 | ([Simeon et al. 2020](#_ENREF_16)) |
| Skin | τ_skin_ | hours | 24 | ([Simeon et al. 2020](#_ENREF_16)) |
| Other tissues | τ_others_ | hour | 0 | ([Simeon et al. 2020](#_ENREF_16)) |
| Organ growth rate |  |  |  |  |
| Brain | k_g, brain_ | hour ^-1^ | 1.33E^-3^ | ([Simeon et al. 2020](#_ENREF_16)) |
| Eyes | k_g, eyes_ | hour ^-1^ | 7.20E^-4^ | ([Simeon et al. 2020](#_ENREF_16)) |
| Gut | k_g, gut_ | hour^-1^ | 1.37E^-3^ | ([Simeon et al. 2020](#_ENREF_16)) |
| Heart | k_g, heart_ | hour ^-1^ | 1.81E^-3^ | ([Simeon et al. 2020](#_ENREF_16)) |
| Liver | k_g, liver_ | hour^-1^ | 3.18E^-4^ | ([Simeon et al. 2020](#_ENREF_16)) |
| Muscle | k_g, muscle_ | hour ^-1^ | 3.04E^-3^ | ([Simeon et al. 2020](#_ENREF_16)) |
| Skeleton | k_g, skeleton_ | hour ^-1^ | 1.09E^-3^ | ([Simeon et al. 2020](#_ENREF_16)) |
| Skin | k_g, skin_ | hour ^-1^ | 8.22E^-4^ | ([Simeon et al. 2020](#_ENREF_16)) |
| Other tissues | k_g, others_ | hour ^-1^ | 2.36E^-3^ | ([Simeon et al. 2020](#_ENREF_16)) |
|  |  |  |  |  |
| Yolk consumption rate | K_d, yolk_ | hour ^-1^ | 3.13E^-4^ | ([Simeon et al. 2020](#_ENREF_16)) |
| Volume of liver cell | V_cell liver_ | µL | 10^-6^ | ([Simeon et al. 2020](#_ENREF_16)) |
| Radius of embryo at initial time | r_embryo,0_ | mm | 0.13 | ([Kimmel et al. 1995](#_ENREF_9)) |
| Radius of yolk at initial time | r_yolk,0_ | mm | 0.4 | ([Kimmel et al. 1995](#_ENREF_9)) |
| Volume of embryo at final time | V_embryo (120hpf)_ | µL | 3.7E^-1^ | ([Simeon et al. 2020](#_ENREF_16)) |
| Organ vol. as fractions of total at 120 hpf |  |  |  |  |
| Others | sc_others (120hpf)_ | - | 1.99E^-1^ | ([Simeon et al. 2020](#_ENREF_16)) |
| Muscle | sc_muscle (120hpf)_ | - | 2.00E^-1^ | ([Simeon et al. 2020](#_ENREF_16)) |
| Arrhenius temperature* | TA | K | 6930 | Estimation based on ([Kimmel et al. 1995](#_ENREF_9)) |
| * The Arrhenius temperature was estimated as described in Kooijman’s Dynamic Energy Budget theory for metabolic organization([Kooijman &Kooijman 2010](#_ENREF_10)) | | | | |

**Table S3.** Prior distributions of BPA partition coefficients from VIVD model and flow rates used in the Bayesian-PBPK model

| Parameter | Values | Comment |
| --- | --- | --- |
| Tissues partition coefficients |  |  |
| yolk / water | 418 | VIVD QSAR model |
| liver / water | 81.1 |  |
| gut / water | 43.5 |  |
| muscle / water | 64.7 |  |
| skeleton / water | 64.7 |  |
| eyes / water | 45.9 |  |
| brain / water | 73.8 |  |
| heart / water | 64.7 |  |
| skin / water | 45.9 |  |
| other tissues / water | 66.9 |  |
|  |  |  |
| Other partition coefficients |  |  |
| polymer / water | 1.74E^-3^ (experiment in plastic device) 1.00E^-6^ (experiment in a low binding material device) | VIVD QSAR model |
| air / water unbound | Nonvolatile (1E^12^) | VIVD QSAR model |
|  |  |  |
| Scaling factor |  |  |
| f_pc_ | [1.00E^-2^ ; 1.00E^1^] |  |
|  |  |  |
| Flow rates |  |  |
| Flow Medium/Plastic (µL/h/mm²) | 1.00E^3^ | Rapid exchange |
| Flow Medium/Air (µL/h/mm²) | 1.00E^3^ | Rapid exchange |
| Flow Post-hatch Medium/Embryo (µL/h/mm²) | [1.00E^-2^ ; 1.00E^2^] |  |
| Flow Pre-hatch Medium/Chorion/Embryo (µL/h) | [1.00E^-2^ ; 1.00E^1^] |  |
|  |  |  |
| Metabolism rate (µmol/h) | [1.00E^-6^ ; 1.00] |  |
| Absorption saturation constant (nmol/µL) | [1.00E^-3^ ; 2.00E^2^] |  |
|  |  |  |

**Table S5.** Physicochemical properties of the bisphenols used in the VIVD model to predict the partition coefficients (unbound fraction was set to 1, assuming no binding in water)

| Chemical | Structure^a^ | Molecular weight (g/mol) | Log Kow | pKa1 (pKa2) at 25°C | Henry's Law constant  atm. m^3^/mol at 25 °C | Water solubility at 25°C |
| --- | --- | --- | --- | --- | --- | --- |
| BPA | 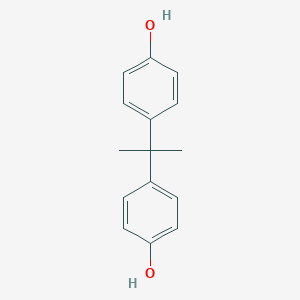 | 228.29 | 3.32 | 9.6 | 4.0E10^-11^ | 300 mg/L |
| BPAF | 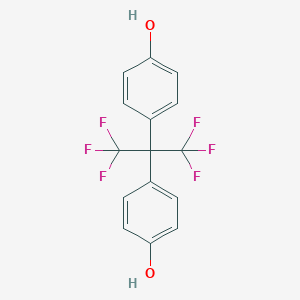 | 336.23 | 4.47 | 9.2 | 5.7E10^-10^ | 4.3 mg/L([Perez et al. 2017](#_ENREF_14)) |
| BPAP | 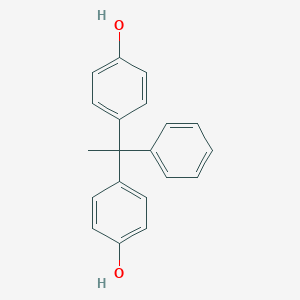 | 290.4 | 4.4 | 10.2([Perez et al. 2017](#_ENREF_14)) | No data | 3.7 mg/L([Perez et al. 2017](#_ENREF_14)) |
| BPF | 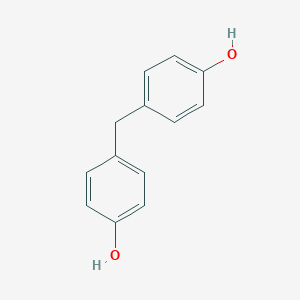 | 200.23 | 2.91 | 7.55 (10.8) | 5.2E10^-12^ | 542.8 mg/L |
| BPB | 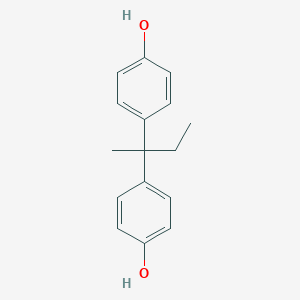 | 242.31 | 4.13 | 10.1 | 1.2E10^-11^ | 29.3 mg/L([Perez et al. 2017](#_ENREF_14)) |
| BPS | 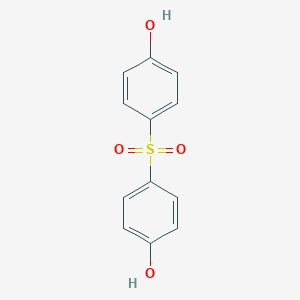 | 250.27 | 1.65 | 8.2 | 2.7E10^-15^ | 3.518 g/L([Perez et al. 2017](#_ENREF_14)) |

Figures and data from Pubchem^®^ (accessed on February 2020) unless stated otherwise

**Table S5.** Mean parameter values used in the sensitivity analysis

| Parameters | Unit | Value | CI 95% | | Distribution | Min | Max | Code names |
| --- | --- | --- | --- | --- | --- | --- | --- | --- |
| f_pc_ |  | 0.365 | 0.276 | 0.522 | Uniform | 0.33 | 0.40 | ffpc |
| Flow Post-hatch Medium/Embryo | (µL/h/mm²) | 8.00 | 5.58 | 12.1 | Uniform | 7.20 | 8.80 | F_embryo |
| Flow Pre-hatch Medium/Chorion/Embryo | (µL/h) | 0.50 | 0.335 | 0.657 | Uniform | 0.45 | 0.54 | F_chorion |
| Flow Medium/Plastic | µL/h/mm² | 1000 | - | - | Uniform | 900 | 1100 | F_plastic |
| Flow Medium/Air | µL/h/mm² | Nonvolatile (1E^-12^) | - | - | - | - | - |  |
| ***partition coefficients*** |  |  |  |  |  |  |  |  |
| yolk / water |  | 418.0 | - | - | Uniform | 376 | 460 | P_yolk_water |
| liver / water |  | 81.1 | - | - | Uniform | 73 | 89 | P_liver_water |
| gut / water |  | 43.5 | - | - | Uniform | 39 | 48 | P_gut_water |
| muscle / water |  | 64.7 | - | - | Uniform | 58 | 71 | P_muscle_water |
| skeleton / water |  | 64.7 | - | - | Uniform | 58 | 71 | P_skeleton_water |
| eyes / water |  | 45.9 | - | - | Uniform | 41 | 50 | P_eyes_water |
| brain / water |  | 73.8 | - | - | Uniform | 66 | 81 | P_brain_water |
| heart / water |  | 64.7 | - | - | Uniform | 58 | 71 | P_heart_water |
| skin / water |  | 45.9 | - | - | Uniform | 41 | 50 | P_skin_water |
| other tissues / water |  | 66.9 | - | - | Uniform | 60 | 74 | P_others_water |
| polymer / water |  | 0.0017 | - | - | Uniform | 0.0016 | 0.0019 | P_pw |
| air / water unbound |  | Nonvolatile (1E^12^) | - | - | - | - | - |  |
|  |  |  |  |  |  |  |  |  |
| Metabolism rate (µL/h) |  | 0.10 |  |  | Uniform | 0.09 | 0.11 | K_met |
| Absorption saturation constant (nmol/µL) |  | 1.00 |  |  | Uniform | 0.9 | 1.1 | Sat_fifty |

**Table S6.** Prior distributions of BPA substitutes partition coefficients from VIVD model, metabolic clearance, and absorption saturation constant used in the Bayesian inference.

| Parameter | BPAF | BPF | BPS | Comment |
| --- | --- | --- | --- | --- |
| Tissues partition coefficients |  |  |  |  |
| yolk / water | 5640 | 162 | 8.80 | VIVD QSAR model |
| liver / water | 1130 | 31.5 | 2.22 |  |
| gut / water | 600 | 17.1 | 1.45 |  |
| muscle / water | 897 | 25.3 | 1.93 |  |
| skeleton / water | 897 | 25.3 | 1.93 |  |
| eyes / water | 632 | 18.1 | 1.64 |  |
| brain / water | 1020 | 28.8 | 2.17 |  |
| heart / water | 897 | 25.3 | 1.93 |  |
| skin / water | 632 | 18.1 | 1.64 |  |
| other tissues / water | 929 | 26.0 | 1.81 |  |
|  |  |  |  |  |
| ~~Other rates~~ |  |  |  |  |
| ~~Metabolic clearance (µL/h)~~ | ~~[1.00E~~^~~-6~~^ ~~; 1.00]~~ | ~~[1.00E~~^~~-6~~^ ~~; 1.00]~~ | ~~[1.00E~~^~~-6~~^ ~~; 1.00]~~ |  |
| ~~Absorption saturation constant (nmol/µL)~~ | ~~[1.00E~~^~~-3~~^ ~~; 20.0]~~ | ~~[1.00E~~^~~-3~~^ ~~; 20.0]~~ | ~~[1.00E~~^~~-3~~^ ~~; 20.0]~~ |  |
|  |  |  |  |  |

**Table S7**. Result of the stepwise approach to select the most appropriate model structure using the BPA dataset.

| f_pc_ | Flow Medium/Embryo (µL/h/mm²) | Flow Medium/Chorion/Embryo (up to 48hpf) | Metabolism rate (µL/h) | Concentration saturation 50 (nmol/µL) | Akaike Information Criteria | Bayesian Information Criteria | Fit^a^ |
| --- | --- | --- | --- | --- | --- | --- | --- |
| X |  |  | X |  | -827 | -823 | Poor |
| X | X |  | X |  | -894 | -888 | Average |
| X | X | X |  |  | -890 | -884 | Good |
| X | X | X | X |  | -889 | -881 | Good |
| X | X | X | X | X | -880 | -871 | Good |

^a^: The fit was considered good when the shape of the kinetic was relevant from a pharmacokinetic point of view

**Table S8.** Unbound Tissues partition coefficients corrected using f_pc_

| Parameter | BPA | BPAF | BPF | BPS |
| --- | --- | --- | --- | --- |
| yolk / water | 144 | 1300 | 275 | 0.998 |
| liver / water | 28.0 | 260 | 53.6 | 0.252 |
| gut / water | 15.0 | 138 | 29.1 | 0.164 |
| muscle / water | 22.3 | 206 | 43.0 | 0.219 |
| skeleton / water | 22.3 | 206 | 43.0 | 0.219 |
| eyes / water | 15.8 | 145 | 30.8 | 0.186 |
| brain / water | 25.4 | 234 | 49.0 | 0.246 |
| heart / water | 22.3 | 206 | 43.0 | 0.219 |
| skin / water | 15.8 | 145 | 30.8 | 0.186 |
| other tissues / water | 23.1 | 213 | 44.2 | 0.205 |

**Table S9.:** Age effect on BPA exposure parameters in eleutheroembryo zebrafish dosed at 1 µM.

| Age when dosing (hpf) | 1 | 12 | 24 | 48 | 72 | 96 | 120 |
| --- | --- | --- | --- | --- | --- | --- | --- |
| t_max_ (h post dose) | 39.5 | 38.9 | 33.5 | 16.3 | 18.3 | 20.0 | 22.0 |
| C_max_ (nmol/mg BW) | 61.2 | 68.9 | 45.2 | 39.2 | 27.3 | 23.0 | 21.4 |

**Table S10.:** Main toxicokinetic exposure parameters in eleutheroembryo zebrafish dosed at 1 µM with either BPA, BPAF, BPF, or BPS.

| Molecule | BPA | BPAF | BPF | BPS |
| --- | --- | --- | --- | --- |
| t_max_ (hpf) | 49.3 | 37.9 | 47.6 | 3.50 |
| C_max_ (nmol/mg BW) | 64.5 | 50.1 | 86.2 | 0.972 |
| AUC_0-120hpf_ (nmol.h/mg BW) | 4910 | 3550 | 7540 | 55.8 |

Additional figures

**
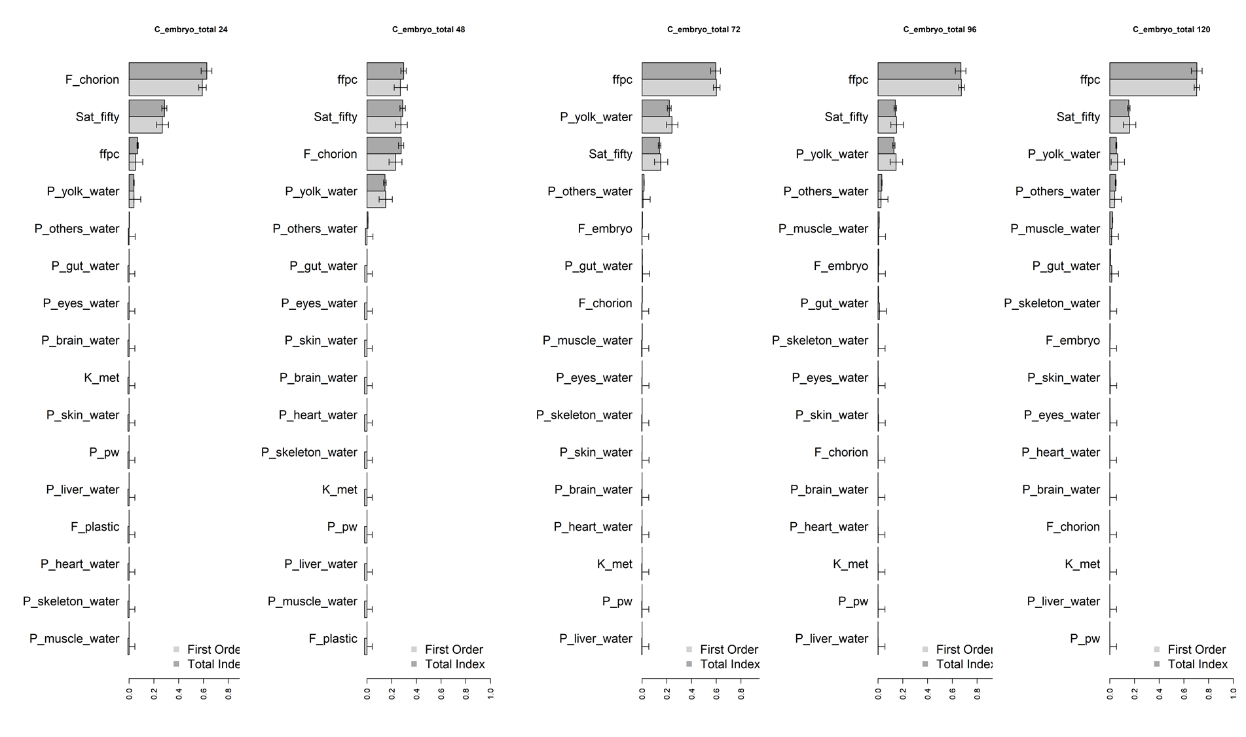
**

**Figure S1A.**: Sensitivity analysis of initial model, including correcting factor of the PC (ffpc), metabolism (Kmet) and saturation process (Sat_Fifty), on total BPA concentrations in ZFE (embryo and yolk), at 24, 48, 72, 96 and 120 hpf (from left to right, respectively). The 15 most influential parameters are represented.

**
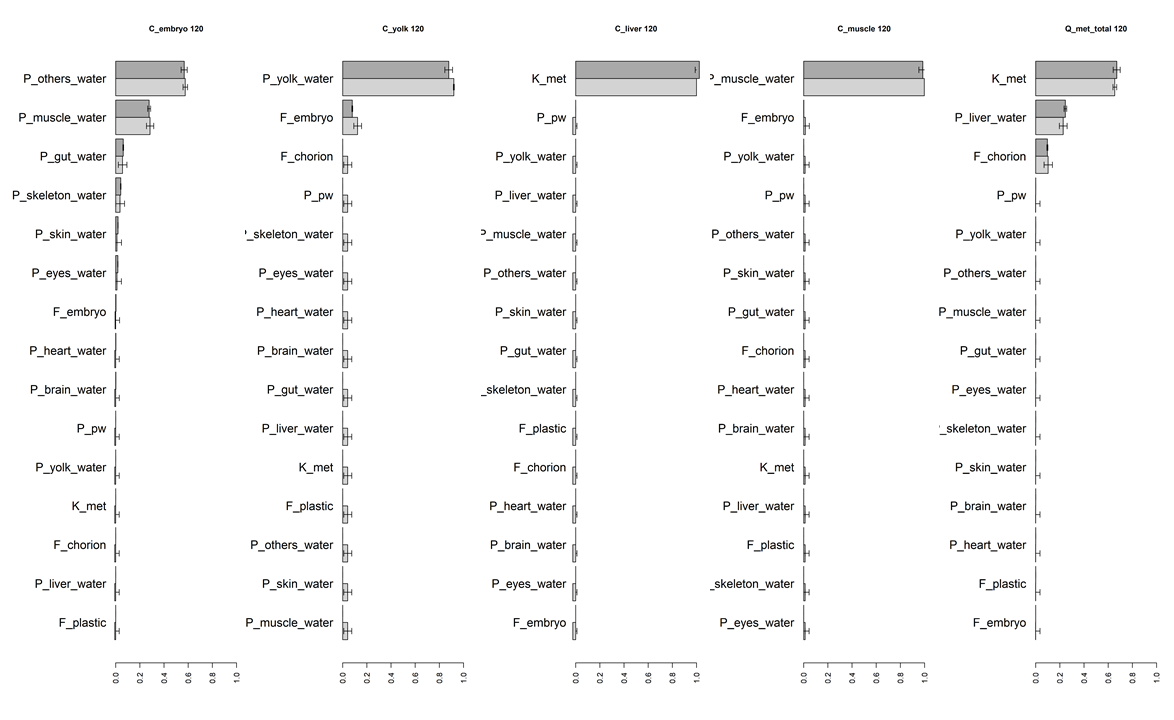
**

**Figure S1B.**: Sensitivity analysis of the final model on BPA concentrations in ZFE embryo, yolk, liver, muscle, Q met at 120 hpf. The 15 most influential parameters are represented.

**
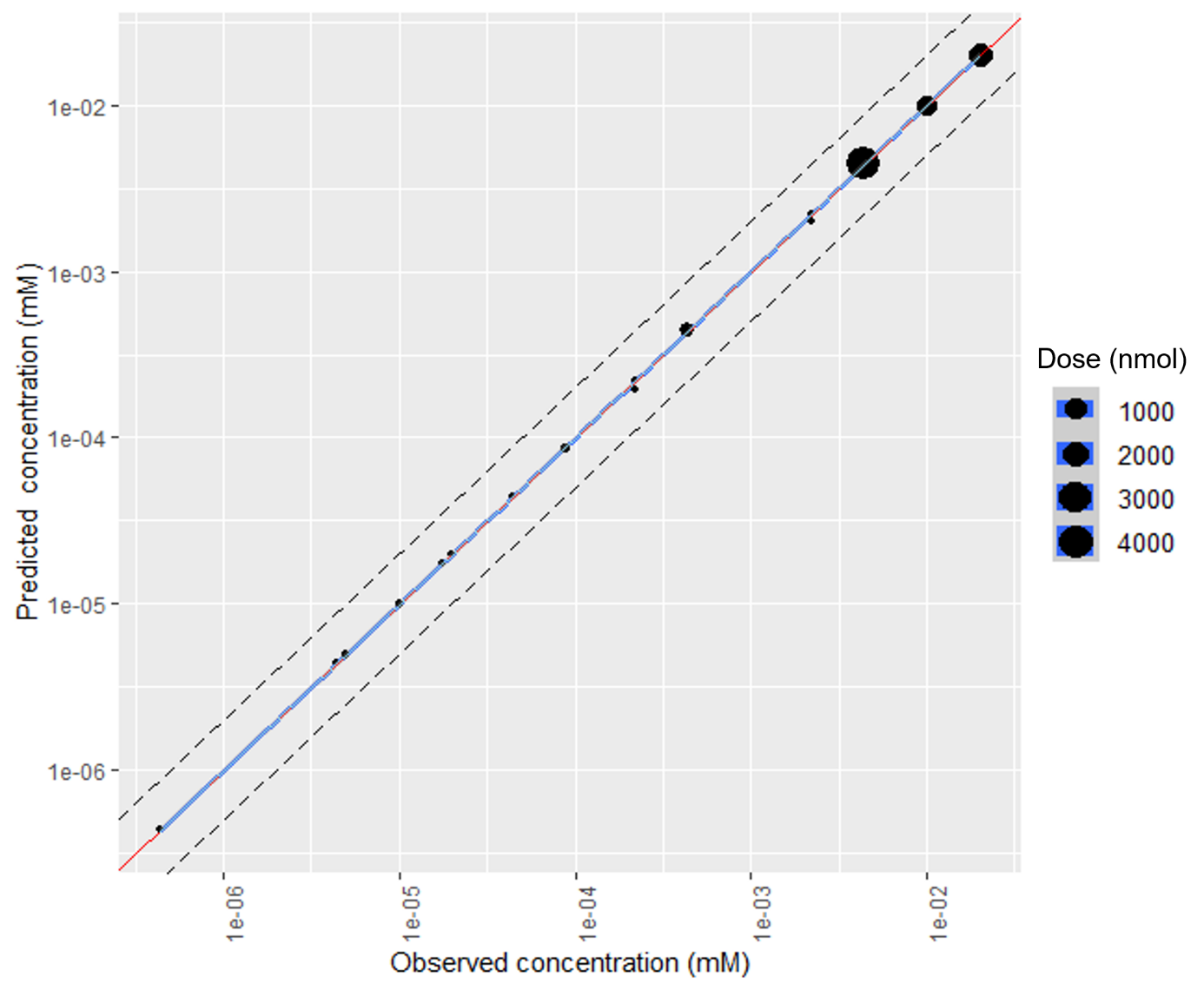
Figure S2.**: Observed *versus* model-predicted Bisphenol A concentrations in the water medium when using the final model (includes the *fpc*, both the pre- and posthatch flows but not metabolic clearance nor absorption saturation parameters). The red and blue lines are the line of identity and semi logistic regression of best fit, respectively. The light grey area defines the 95% credibility interval of the model, the long-dashed line is the 2-fold error.
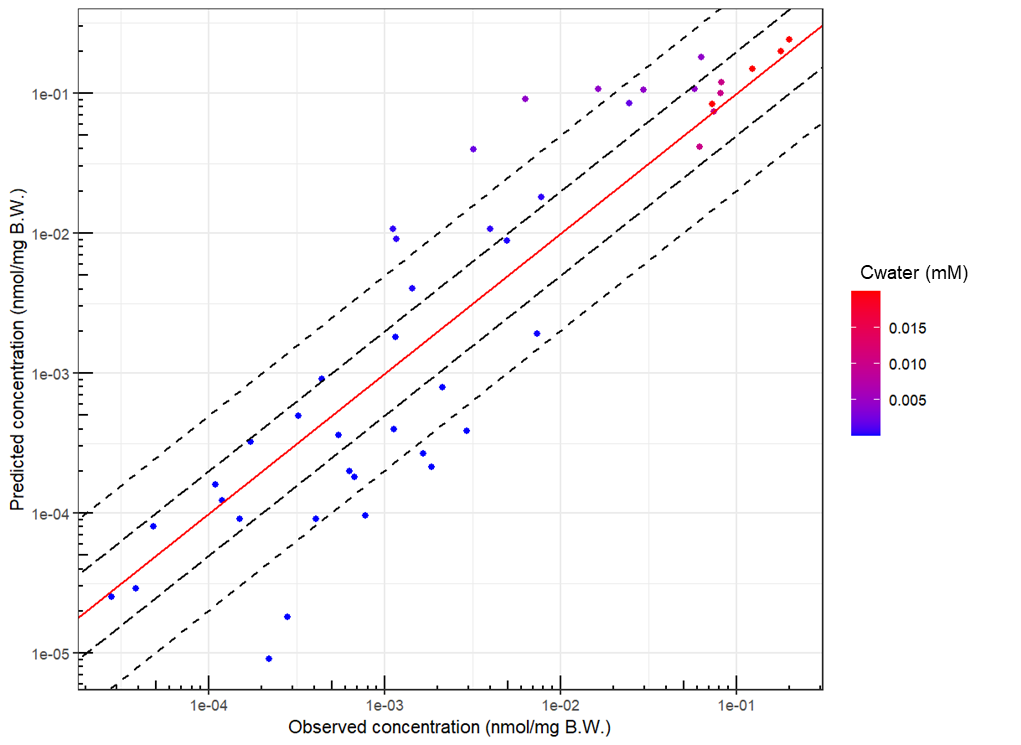
**Figure S3.** Bisphenol A observed concentrations *versus* model-predicted concentrations in the zebrafish eleutheroembryo when using the final model. The red line is the line of identity. The long dashed and dashed lines are the 2- and 5-fold errors, respectively.


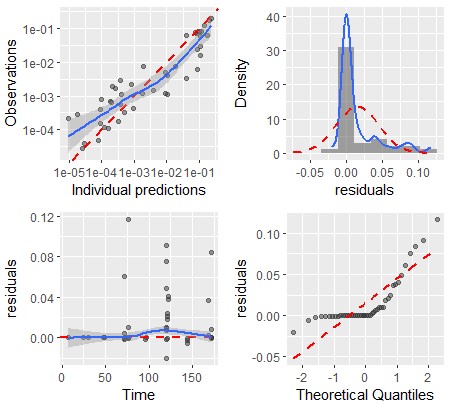


**Figure S4.**: Diagnostic plots of the final model.


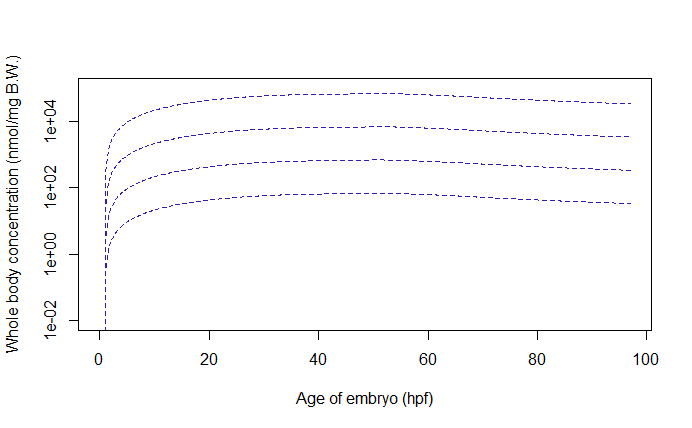
**Figure S5.**: Simulations of the impact of the water concentration on BPA exposure in 1-hpf aged eleutheroembryo zebrafish (1, 10, 100, and 1,000µM BPA in 2 mL of water, semilogarithmic scale)
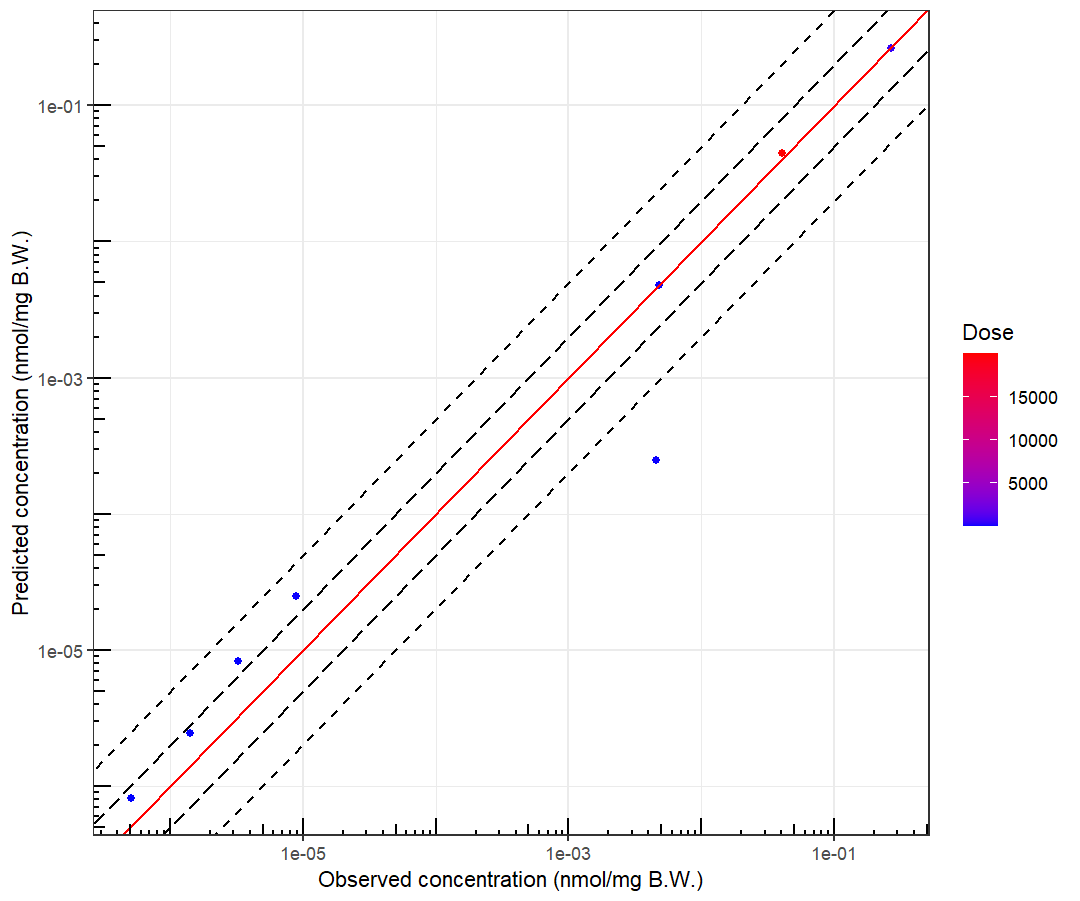
Figure S6. Observed versus model-predicted concentrations of BPAF, BPF, and BPS in the zebrafish eleutheroembryo in water for Bisphenol AF, F, and S when using the PBPK model. The red line is the line of identity. The long dashed and dashed lines are the 2- and 5-fold errors, respectively.


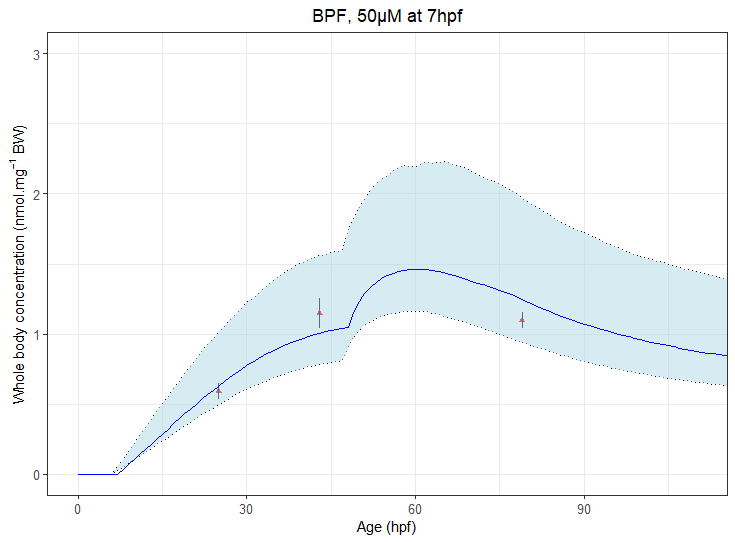


**Figure S7.** Comparison of the model predicted concentrations (including saturation processes, full curve) with the observed concentrations (triangles) with standard deviation (error bars) from the BPF data set (the grey area is the 95% credibility interval).


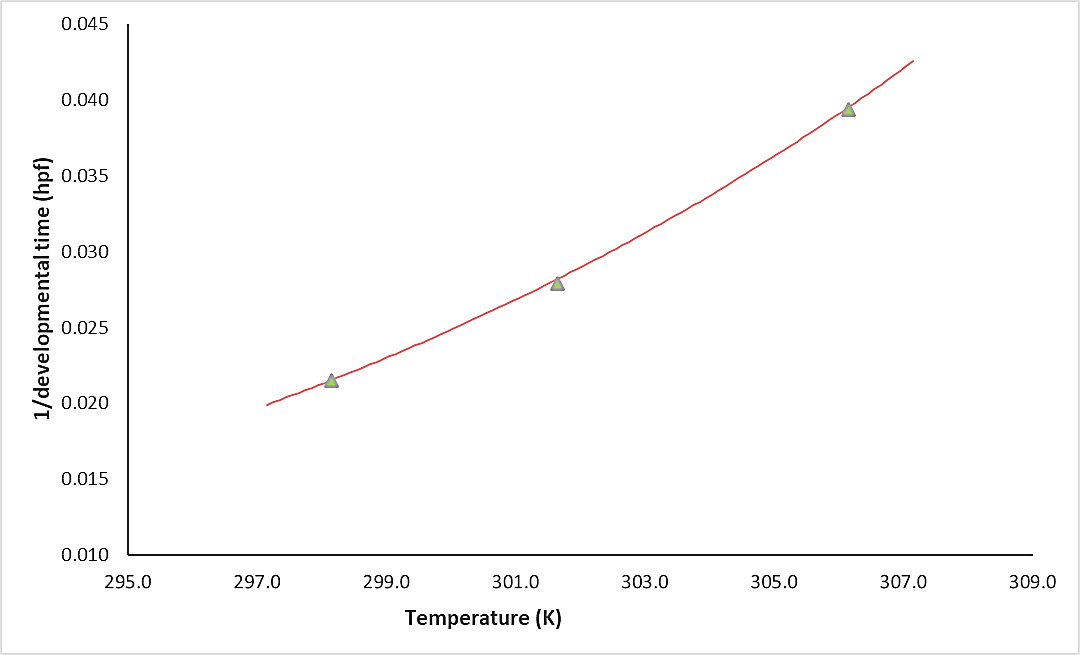


**Figure S8.:** Temperature effect on the inverse of the developmental time of the stage “Hatching” described by [Kimmel et al. (1995)](#_ENREF_9). Green dots are observations reported by [Kimmel et al. (1995)](#_ENREF_9) and the red line is the least-squares regression curve of the Arrhenius relationship. TR was fixed to 298.15K (organ growth was calibrated to this temperature by Siméon et al. (2020)) and Arrhenius temperature (TA) was estimated at 6930K.

Model code

Code of structural model (MCSim)

# Zebra fish embryo PK model

# Dynamic version, with linear metabolism

# Frederic Bois Reproductive Toxicology 2020

# Remy Beaudouin, Jan 2020

# Pierre-A Billat, Apr 2021

# Units:

# quantities: nmol

# volumes: mm3

# concentrations: nmol/mg BW

# time: hours

=================================================================

# V1 : Basic dynamic model including flux without medium saturation

# V2 : Addition of a Flow through the chorion (up to 48h), temperature effect.

################ Variables ################

States = { Q_water, # Quantity in water (nmoles)

Q_water_add,

Q_air, # ~ in air

Q_polymer, # ~ bound on polymer

Q_met, # ~ of metabolites in system

Q_yolk, # ~ per embryo in yolk

Q_liver, # ~ in liver

Q_gut, # ~ in digestive tract

Q_muscle, # ~ in muscles

Q_skeleton, # ~ in skeleton

Q_eyes, # ~ in eyes

Q_brain, # ~ in brain

Q_heart, # ~ in heart

Q_skin, # ~ in skin pigmented cells

Q_others # ~ in other tissues

};

Inputs = { Event_labile,

Event_labile_bis,

Event_labile_air,

N_embryo} # Number of embryos per well;

Outputs = { # Concentration in embryo (nmol/mg wet BW)

C_yolk, # Concentration in yolk

C_liver, # ~ in liver (metabolizes)

C_gut, # ~ in digestive tract

C_muscle, # ~ in muscles

C_skeleton, # ~ in skeleton

C_eyes, # ~ in eyes

C_brain, # ~ in brain

C_heart, # ~ in heart

C_skin, # ~ in skin pigmented cells

C_others, # ~ in other tissues

C_embryo, # ~ in embryo, not including yolk

C_embryo_total, # ~ in embryo, including yolk

# Quantity in embryo

Q_embryo, # Quantity in embryo, not including yolk

Q_embryo_total, # Quantity in N embryo, including yolk

Q_met_total, # Quantity of metabolites due to the N embryo

# Embryo biometric variables

V_embryo, # volume per embryo (mm3)

V_yolk, # volume of yolk (mm3)

V_embryo_total, # Sum of embryo and yolk volumes (mm3)

V_water_embryo,

V_liver, # ~ liver

V_gut, # ~ gut

V_muscle, # ~ muscles

V_skeleton, # ~ skeleton

V_eyes, # ~ eyes

V_brain, # ~ brain

V_heart, # ~ heart

V_skin, # ~ skin

V_others, # ~ others embryonic tissues

# System related variables

V_air, # Volume of air in the system

S_p_w, # Surface area of water in contact with wall (mm2)

C_water, # Concentration of parent in water (nmol/mm3)

C_air, # Concentration of parent in air

V_content,

K_polymer,

F_yolk,

F_liver,

F_gut,

F_skeleton,

F_eyes,

F_brain,

F_heart,

F_skin,

F_others ,

F_muscle ,

# Coumpound related variables

Q_check, # Quantity mass balance check, per embryo

Q_water_supp

};

################ Parameters ################

Pi = 3.14159265358979323846; # Pi Number

################ System Physical Parameters ################

V_well; # Volume of a culture well (mm3)

D_well; # Diameter of a well (mm)

V_water; # volume of culture medium at time 0 (mm3)

S_a_w; # surface area of water in contact with air (mm2)

Temperature = 301.15; # experimental temperature (degrees K) = 28°C + 273.15

TR = 298.15 ; # Reference temperatures in °K (25°C)

TA = 6930 ; # Arrhenius temperature in Kelvin

A_FC; # Arrhenius temperatures fonction

T_fec = 0; # time of egg fecundation (can be > t0 experiment)

############### Exposure-designed Parameters ###############

Dose; # Total dose of parent (nmol) in total water at start

############### Substance specific Parameters ###############

fui; # Fraction unionized in water

F_polymer ; # (in uL/h/mm2) adsorption rates for non-specific binding on polymer;

F_air ; # flow (in uL/h/mm2) evaporation and absorption rates water/air;

F_embryo; # flow (in uL/h) exchange rates water/embryo;

F_chorion; # Fraction of flow exchange (in %) rates water/ embryo through chorionated embryo;

Michaelis = 0; # Flag for Michaelis-Menten vs linear metabolism (0 = linear)

K_met; # Metabolic clearance per liver cell, if linear metabolism, uL/h

Vmax; # Maximum formation rate of metabolite, if saturable metabolism, nmol/h

Km; # Michaelis-Menten constant, if saturable metabolism, nmol/uL

Sat_fifty; # Saturation C50 (nmol/uL)

# Parameters recomputed in initialize section: must be specified in the input file

P_pw; # Polymer / water partition coefficient

P_aw; # Air / water partition coefficient

#P_x:w = partition coefficients ORGAN/MEDIUM.

P_yolk_water; # yolk partition coefficient / Medium unbound

P_liver_water; # liver partition coefficient / Medium unbound

P_gut_water; # ~ partition coefficient / Medium unbound

P_muscle_water; # ~ partition coefficient / Medium unbound

P_skeleton_water; # ~ partition coefficient / Medium unbound

P_eyes_water; # ~ partition coefficient / Medium unbound

P_brain_water; # ~ partition coefficient / Medium unbound

P_heart_water; # ~ partition coefficient / Medium unbound

P_skin_water; # ~ partition coefficient / Medium unbound

P_others_water; # ~ partition coefficient / Medium unbound

################# Embryo biometrics #################

# Volume of the embryo without yolk at 0 and 120 hpf (uL)

V_embryo_0 = 0.005;

V_embryo_120 = 0.371;

V_cell_liver = 3.4e-6; # volume of a liver cell (uL)

# Starting times for organ growth (hours) (from K. Brotzmann)

tau_liver = 16 ;

tau_gut = 10 ;

tau_skeleton = 48 ;

tau_eyes = 11 ;

tau_brain = 9 ;

tau_heart = 30 ;

tau_skin = 24 ;

tau_muscle = 60 ;

tau_others = 0 ;

# Yolk parameters

K_d_yolk ;

V_yolk_0 = 0.207;

# Organ growth rates (1/h) as fractions of total embryo (without yolk) at 120 hpf

K_g_liver = (60* 5.29509e-06);

K_g_gut = (60* 2.2779e-05);

K_g_skeleton = (60* 1.82294e-05);

K_g_eyes = (60* 1.20414e-05);

K_g_brain = (60* 2.22253e-06);

K_g_heart = (60* 3.01003e-06);

K_g_skin = (60* 1.3672e-05);

K_g_others = (60* 3.92738e-05);

K_g_muscle = (60* 5.07074e-05);

################## Other parameters ##################

sigma; # Statistical parameter

ffpc; # Fudge factor for partition coefficients

################ Initialization ################

# -------------------------------------------

Initialize { # assumes that start time is zero, should be revised if not

# Arrhenius temperatures function

A_FC = exp ( (TA / TR) - (TA / Temperature) ) ;

K_d_yolk = (0.000313*60) * A_FC; # Yolk consumption rate constant (1/h)

# Adjusted starting times for organ growth (hours)

tau_liver = tau_liver /A_FC;

tau_gut = tau_gut / A_FC;

tau_skeleton = tau_skeleton / A_FC;

tau_eyes = tau_eyes / A_FC;

tau_brain = tau_brain / A_FC;

tau_heart = tau_heart / A_FC;

tau_skin = tau_skin / A_FC;

tau_muscle = tau_muscle / A_FC;

tau_others = tau_others / A_FC;

# Adjusted partition coefficients

P_yolk_water = P_yolk_water * ffpc;

P_liver_water = P_liver_water * ffpc;

P_gut_water = P_gut_water * ffpc;

P_muscle_water = P_muscle_water * ffpc;

P_skeleton_water = P_skeleton_water * ffpc;

P_eyes_water = P_eyes_water * ffpc;

P_brain_water = P_brain_water * ffpc;

P_heart_water = P_heart_water * ffpc;

P_skin_water = P_skin_water * ffpc;

P_others_water = P_others_water * ffpc;

P_aw = P_aw * ffpc;

# Quantity in water without embryo in the well (nmol)

Q_water = 0;

Q_polymer = 0;

Q_air = 0;

S_a_w = Pi * 0.25 * D_well * D_well; # Surface area of water in contact with recipient area(nm2)

S_p_w = 4 * V_water / D_well + Pi * 0.25 * D_well * D_well;

# Total volume of one embryo at start (uL)

V_embryo_total = V_yolk_0 + V_embryo_0;

# Total volume of water + embryo (uL)

V_content = V_water + ( V_embryo_total * N_embryo);

# Volume of air (uL) in the (sealed) well headspace, per embryo

V_air = V_well - V_content;}

################ Dynamics ################

Dynamics {

# Scaling coefficients of organ volumes (change with time)

sc_liver = (t < (tau_liver + T_fec) ? 0.0 : (exp(K_g_liver * A_FC * (t - tau_liver - T_fec )) - 1));

sc_gut = (t < (tau_gut + T_fec) ? 0.0 : (exp(K_g_gut * A_FC * (t - tau_gut - T_fec )) – 1));

sc_skeleton = (t < (tau_skeleton + T_fec) ? 0.0 : (exp(K_g_skeleton * A_FC * (t - tau_skeleton - T_fec )) - 1));

sc_eyes = (t < (tau_eyes + T_fec) ? 0.0 : (exp(K_g_eyes * A_FC * (t - tau_eyes - T_fec )) - 1));

sc_brain = (t < (tau_brain + T_fec) ? 0.0 : (exp(K_g_brain * A_FC * (t - tau_brain - T_fec )) - 1));

sc_heart = (t < (tau_heart + T_fec) ? 0.0 : (exp(K_g_heart * A_FC * (t - tau_heart - T_fec )) - 1));

sc_skin = (t < (tau_skin + T_fec) ? 0.0 : (exp(K_g_skin * A_FC * (t - tau_skin - T_fec )) - 1));

sc_muscle = (t < (tau_muscle + T_fec) ? 0.0 : (exp(K_g_muscle * A_FC * (t - tau_muscle - T_fec )) - 1));

sc_others = (t < (tau_others + T_fec) ? 0.0 : (exp(K_g_others * A_FC * (t - tau_others - T_fec )) - 1));

# Organ volumes (uL):

V_liver = V_embryo_120 * sc_liver;

V_gut = V_embryo_120 * sc_gut;

V_skeleton = V_embryo_120 * sc_skeleton;

V_eyes = V_embryo_120 * sc_eyes;

V_brain = V_embryo_120 * sc_brain;

V_heart = V_embryo_120 * sc_heart;

V_skin = V_embryo_120 * sc_skin;

V_muscle = V_embryo_120 * sc_muscle;

V_others = (t < T_fec ? 0.0 : V_embryo_120 * sc_others + V_embryo_0 );

# Yolk volume dynamics (uL)

V_yolk = (t < T_fec ? 0.0 : V_yolk_0 * exp(- K_d_yolk * (t- T_fec) ) ); # Volume of yolk (uL), decreases approximately exponentially with time

# Embryo biometrics dynamics (uL)

V_embryo = V_liver + V_gut + V_skeleton + V_eyes + V_brain + V_heart + V_skin + V_others + V_muscle ; # Volume of the embryo without yolk (uL)

V_embryo_total = V_yolk + V_embryo; # Volume of the embryo (uL)

# Flow changes with time (including chorion membrane (up to 48H))

F_embryo_dyn = F_chorion*(1-X_F) + F_embryo*pow(V_embryo_total, 0.667)*X_F ;

# Organ flow (V_i / V_embryo_total ~ S_i / S_embryo_total)

F_yolk = (V_yolk > 1E-12 ? (F_embryo_dyn * V_yolk ) : 1E-12 );

F_liver = (V_liver > 1E-12 ? (F_embryo_dyn * V_liver ) : 1E-12 );

F_gut = (V_gut > 1E-12 ? (F_embryo_dyn * V_gut ) : 1E-12 );

F_skeleton = (V_skeleton > 1E-12 ? (F_embryo_dyn * V_skeleton) : 1E-12 );

F_eyes = (V_eyes > 1E-12 ? (F_embryo_dyn * V_eyes ) : 1E-12 );

F_brain = (V_brain > 1E-12 ? (F_embryo_dyn * V_brain ) : 1E-12 );

F_heart = (V_heart > 1E-12 ? (F_embryo_dyn * V_heart ) : 1E-12 );

F_skin = (V_skin > 1E-12 ? (F_embryo_dyn * V_skin ) : 1E-12 );

F_others = (V_others > 1E-12 ? (F_embryo_dyn * V_others ) : 1E-12 );

F_muscle = (V_muscle > 1E-12 ? (F_embryo_dyn * V_muscle ) : 1E-12 );

F_yolk = (V_yolk > 1E-12 ? (F_embryo_dyn * V_yolk ) : 1E-12 );

# Organ concentrations (V can be null)

C_yolk = ( V_yolk > 1E-12 ? Q_yolk / V_yolk : 1E-12 ) ;

C_liver = ( V_liver > 1E-12 ? Q_liver / V_liver : 1E-12 ) ;

C_gut = ( V_gut > 1E-12 ? Q_gut / V_gut : 1E-12 ) ;

C_muscle = ( V_muscle > 1E-12 ? Q_muscle / V_muscle : 1E-12 ) ;

C_skeleton = ( V_skeleton > 1E-12 ? Q_skeleton / V_skeleton : 1E-12 ) ;

C_eyes = ( V_eyes > 1E-12 ? Q_eyes / V_eyes : 1E-12 ) ;

C_brain = ( V_brain > 1E-12 ? Q_brain / V_brain : 1E-12 ) ;

C_heart = ( V_heart > 1E-12 ? Q_heart / V_heart : 1E-12 ) ;

C_skin = ( V_skin > 1E-12 ? Q_skin / V_skin : 1E-12 ) ;

C_others = ( V_others > 1E-12 ? Q_others / V_others : 1E-12 ) ;

############### Water dynamics ###############

V_content = V_water + ( V_embryo_total * N_embryo); # Total volume of water + embryo (uL) only V_embryo_total is assumed to change with time

S_p_w = 4 * V_content / D_well + Pi * 0.25 * D_well * D_well; # Surface area of water in contact with plastic, with the embryo in the well (mm2)

K_plastic = F_plastic /( P_pw * S_p_w ); # (in 1/h.mm2) desorption rates for non-specific binding on plastic;

C_water = Q_water / V_water; # Concentration unbound in water (nmol/uL)

# Compute dimension PER EMBRYO

V_water_embryo = ( N_embryo > 0.1 ? V_water / N_embryo : 1E-12);

Q_water_e = ( N_embryo > 0.1 ? Q_water / N_embryo : 1E-12); # Quantity available per embryo in water (nmol/uL)

C_water_e = ( N_embryo > 0.1 ? Q_water_e /V_water_embryo : 1E-12 ); # concentration per embryo in water (nmol/uL). If Q is not limiting, C_water = C_water_e

V_air = V_well - V_content; # Volume of air (uL) in head-space

C_air = Q_air / V_air; # Concentration (nmol/uL)

Saturation = 1 / ( 1 + C_water_e / Sat_fifty);# ---> term to be used to model saturation

dt(Q_water) = F_air * S_a_w * C_air / P_aw - F_air * S_a_w * C_water * fui #air exchanges

+ K_plastic * S_p_w * Q_plastic - F_plastic * S_p_w * C_water # plastic exchanges

+ N_embryo * F_yolk * (C_yolk / P_yolk_water - C_water_e * Saturation) # yolk

+ N_embryo * F_liver * (C_liver / P_liver_water - C_water_e * Saturation) # liver

+ N_embryo * F_gut * (C_gut / P_gut_water - C_water_e * Saturation) # gut

+ N_embryo*F_muscle*(C_muscle/P_muscle_water- C_water_e*Saturation) # muscle

+N_embryo*F_skeleton*(C_skeleton/P_skeleton_water - C_water_e*Saturation) #skeleton

+ N_embryo * F_eyes * (C_eyes / P_eyes_water - C_water_e * Saturation ) # eyes

+ N_embryo * F_brain * (C_brain / P_brain_water - C_water_e *Saturation) # brain

+ N_embryo * F_heart * (C_heart / P_heart_water - C_water_e *Saturation) # heart

+ N_embryo * F_skin * (C_skin / P_skin_water - C_water_e *Saturation) # skin

+ N_embryo * F_others *(C_others/ P_others_water - C_water_e * Saturation); # others

### Air quantity dynamics ##################

dt(Q_air)=F_air * S_a_w * C_water * fui - F_air * S_a_w * C_air / P_aw; # quantity (nmol)

### Plastic quantity dynamics #################

dt(Q_plastic) = F_plastic * S_p_w * C_water - K_plastic * S_p_w * Q_plastic; # Quantity on plastic (nmol)

###### Quantity and concentration in organs, null before organogenesis ######

# Quantity and concentration in yolk (nmol/uL)

dt(Q_yolk) = F_yolk * (C_water_e * Saturation - C_yolk / P_yolk_water );

# Linear metabolism in embryo, per embryo

N_cells = V_liver / V_cell_liver; # null before liver organogenesis

dt(Q_met) = (Michaelis > 0.5 ?

N_cells * C_liver * Vmax / (Km + C_liver) : # MM

N_cells * C_liver * K_met); # linear

# Quantity and concentration in liver (nmol/mg), null before organogenesis

dt(Q_liver) = F_liver * (C_water_e * Saturation - (C_liver/ P_liver_water ) - dt(Q_met));

# Quantity and concentration in gut (nmol/mg), null before organogenesis

dt(Q_gut) = F_gut * (C_water_e * Saturation- C_gut / P_gut_water );

# Quantity and concentration in muscle (nmol/mg), null before organogenesis

dt(Q_muscle) = F_muscle * (C_water_e * Saturation - C_muscle / P_muscle_water ) ;

# Quantity and concentration in skeleton (nmol/mg), null before organogenesis

dt(Q_skeleton) = F_skeleton * (C_water_e *Saturation - C_skeleton / P_skeleton_water ) ;

# Quantity and concentration in eyes (nmol/mg), null before organogenesis

dt(Q_eyes) = F_eyes * ( C_water_e * Saturation - C_eyes / P_eyes_water) ;

# Quantity and concentration in brain (nmol/mg), null before organogenesis

dt(Q_brain) = F_brain * ( C_water_e * Saturation - C_brain / P_brain_water ) ;

# Quantity and concentration in heart (nmol/mg), null before organogenesis

dt(Q_heart) = F_heart *( C_water_e * Saturation- C_heart / P_heart_water ) ;

# Quantity and concentration in skin (nmol/mg), null before organogenesis

dt(Q_skin) = F_skin * ( C_water_e * Saturation- C_skin / P_skin_water );

# Quantity and concentration in gut (nmol/mg), null before organogenesis

dt(Q_others) = F_others * ( C_water_e * Saturation - C_others / P_others_water );

### Quantity and concentration in embryo (nmol/uL) ##################

dt(Q_water_add) = 0;

Q_embryo = Q_liver + Q_gut + Q_muscle + Q_skeleton + Q_eyes + Q_brain + Q_heart + Q_skin + Q_others;

C_embryo = (t < T_fec ? 1E-10 : Q_embryo / V_embryo );

Q_embryo_total = N_embryo * (Q_embryo + Q_yolk);

Q_met_total = N_embryo * Q_met ;

C_embryo_total = (t < T_fec ? 1E-10 : (Q_embryo + Q_yolk) / V_embryo_total );}

CalcOutputs {

Q_water_supp = Q_water + Q_embryo_total + Q_air + Q_plastic + Q_met_total - Q_water_add;

# Safeguards against negative of null values

C_embryo = (C_embryo > 0 ? C_embryo : 1E-12);

C_embryo_total = (C_embryo_total > 0 ? C_embryo_total : 1E-12);

C_water = (C_water > 0 ? C_water : 1E-12);

C_brain = (C_brain > 0 ? C_brain : 1E-12);

Q_check = (Q_water_add + Q_water_supp) - ( Q_water + Q_embryo_total + Q_air + Q_plastic + Q_met_total );}

End.

References

Brown AR, Green JM, Moreman J, Gunnarsson LM, Mourabit S, Ball J, Winter MJ, Trznadel M, Correia A, Hacker C, Perry A, Wood ME, Hetheridge MJ, Currie RA, Tyler CR (2019): Cardiovascular Effects and Molecular Mechanisms of Bisphenol A and Its Metabolite MBP in Zebrafish. Environmental science & technology 53, 463-474

Fisher C, Siméon S, Jamei M, Gardner I, Bois YF (2018): VIVD: Virtual in vitro distribution model for the mechanistic prediction of intracellular concentrations of chemicals in in vitro toxicity assays. Toxicology in Vitro

Fu J, Guo Y, Yang L, Han J, Zhou B (2020): Nano-TiO2 enhanced bioaccumulation and developmental neurotoxicity of bisphenol a in zebrafish larvae. Environmental research 187, 109682

Gelman A, Rubin DB (1996): Markov chain Monte Carlo methods in biostatistics. Statistical methods in medical research 5, 339-55

Gibert Y, Sassi-Messai S, Fini JB, Bernard L, Zalko D, Cravedi JP, Balaguer P, Andersson-Lendahl M, Demeneix B, Laudet V (2011): Bisphenol A induces otolith malformations during vertebrate embryogenesis. BMC developmental biology 11, 4

Grech A, Tebby C, Brochot C, Bois FY, Bado-Nilles A, Dorne JL, Quignot N, Beaudouin R (2019): Generic physiologically-based toxicokinetic modelling for fish: Integration of environmental factors and species variability. Science of the Total Environment 651, 516-531

Henn K, Braunbeck T (2011): Dechorionation as a tool to improve the fish embryo toxicity test (FET) with the zebrafish (Danio rerio). Comp Biochem Phys C 153, 91-98

Kim SS, Hwang KS, Yang JY, Chae JS, Kim GR, Kan H, Jung MH, Lee HY, Song JS, Ahn S, Shin DS, Lee KR, Kim SK, Bae MA (2020): Neurochemical and behavioral analysis by acute exposure to bisphenol A in zebrafish larvae model. Chemosphere 239, 124751

Kimmel CB, Ballard WW, Kimmel SR, Ullmann B, Schilling TF (1995): Stages of Embryonic-Development of the Zebrafish. Dev Dynam 203, 253-310

Kooijman B, Kooijman S (2010): Dynamic energy budget theory for metabolic organisation. Cambridge university press

Le Fol V, Brion F, Hillenweck A, Perdu E, Bruel S, Ait-Aissa S, Cravedi JP, Zalko D (2017): Comparison of the In Vivo Biotransformation of Two Emerging Estrogenic Contaminants, BP2 and BPS, in Zebrafish Embryos and Adults. International journal of molecular sciences 18

Moreman J, Lee O, Trznadel M, David A, Kudoh T, Tyler CR (2017): Acute Toxicity, Teratogenic, and Estrogenic Effects of Bisphenol A and Its Alternative Replacements Bisphenol S, Bisphenol F, and Bisphenol AF in Zebrafish Embryo-Larvae. Environmental science & technology 51, 12796-12805

Moreman J, Takesono A, Trznadel M, Winter MJ, Perry A, Wood ME, Rogers NJ, Kudoh T, Tyler CR (2018): Estrogenic Mechanisms and Cardiac Responses Following Early Life Exposure to Bisphenol A (BPA) and Its Metabolite 4-Methyl-2,4-bis( p-hydroxyphenyl)pent-1-ene (MBP) in Zebrafish. Environmental science & technology 52, 6656-6665

Perez RA, Albero B, Ferriz M, Tadeo JL (2017): Rapid multiresidue determination of bisphenol analogues in soil with on-line derivatization. Analytical and bioanalytical chemistry 409, 4571-4580

Saltelli A, Chan K, Scott EM (2008): Sensitivity Analysis, New York

Simeon S, Brotzmann K, Fisher C, Gardner I, Silvester S, Maclennan R, Walker P, Braunbeck T, Bois FY (2020): Development of a generic zebrafish embryo PBPK model and application to the developmental toxicity assessment of valproic acid analogs. Reprod Toxicol 93, 219-229

Sobol IM, Tarantola S, Gatelli D, Kucherenko SS, Mauntz W (2007): Estimating the approximation errors when fixing unessential factors in global sensitivity analysis. Reliability Engineering & System Safety 92, 957-960

Souder JP, Gorelick DA (2018): Assaying uptake of endocrine disruptor compounds in zebrafish embryos and larvae. Comparative biochemistry and physiology. Toxicology & pharmacology : CBP 208, 105-113

van de Schoot R, Depaoli S, King R, Kramer B, Märtens K, Tadesse MG, Vannucci M, Gelman A, Veen D, Willemsen J, Yau C (2021): Bayesian statistics and modelling. Nature Reviews Methods Primers 1, 1

Wu M, Pan C, Chen Z, Jiang L, Lei P, Yang M (2017): Bioconcentration pattern and induced apoptosis of bisphenol A in zebrafish embryos at environmentally relevant concentrations. Environmental science and pollution research international 24, 6611-6621

Yang J, Zhong W, Chen P, Zhang Y, Sun B, Liu M, Zhu Y, Zhu L (2019): Graphene oxide mitigates endocrine disruption effects of bisphenol A on zebrafish at an early development stage. The Science of the total environment 697, 134158

Zhang DH, Zhou EX, Yang ZL (2017): Waterborne exposure to BPS causes thyroid endocrine disruption in zebrafish larvae. PloS one 12, e0176927
